# Divergent transcriptional programs and constrained metabolic states reveal selective metabolic dependencies in chemoresistant luminal breast cancer

**DOI:** 10.20517/cdr.2026.57

**Published:** 2026-08-28

**Authors:** Melis Poyraz, Buse Sari, Jérôme Ambroise, Melanie Spears, Martin Michaelis, Jindrich Cinatl Jr., Romain Boidot, Kristian Serafimov, Olivier Feron, François P. Duhoux, Manon Desgres, Cyril Corbet

**Affiliations:** ^1^Pole of Pharmacology and Therapeutics (FATH), Institut de Recherche Expérimentale et Clinique (IREC), UCLouvain, Brussels 1200, Belgium.; ^2^Centre des Technologies Moléculaires Appliquées (CTMA), Institut de Recherche Expérimentale et Clinique (IREC), UCLouvain, Brussels 1200, Belgium.; ^3^Diagnostic Development, Ontario Institute for Cancer Research, Toronto M5G 1M1, Canada.; ^4^Department of Laboratory Medicine and Pathobiology, University of Toronto, Toronto M5S 3K9, Canada.; ^5^School of Natural Sciences, Stacey Building, University of Kent, Canterbury CT2 7NJ, UK.; ^6^Dr Petra Joh-Research Institute, Frankfurt am Main 60528, Germany.; ^7^Université Bourgogne Europe, Centre Georges-François Leclerc, Unicancer, Unit of Molecular Biology, Department of Biology and Pathology of Tumors, ICMUB UMR CNRS 6302, Dijon 21000, France.; ^8^IREC Metabolomics Core, UCLouvain, Brussels 1200, Belgium.; ^9^WEL Research Institute, Wavre 1300, Belgium.; ^10^Pole of Molecular Imaging, Radiotherapy and Oncology (MIRO), Institut de Recherche Expérimentale et Clinique (IREC), UCLouvain, Brussels 1200, Belgium.; ^11^Institut Roi Albert II, Department of Medical Oncology, Cliniques universitaires Saint-Luc, Brussels 1200, Belgium.; ^12^These authors equally supervised the work.

**Keywords:** Breast cancer, HR+/HER2- disease, neoadjuvant chemotherapy, chemoresistance, taxane, anthracycline, polyamine metabolism, metabolic plasticity

## Abstract

**Aim:** Resistance to neoadjuvant chemotherapy remains a major challenge in hormone receptor-positive/human epidermal growth factor receptor 2-negative (HR+/HER2-) breast cancer (BC). Although anthracyclines and taxanes constitute the standard sequential regimen, the metabolic adaptations accompanying resistance to these agents remain poorly defined. We investigated whether chemoresistance is associated with shared or drug-specific metabolic alterations.

**Methods:** Parental MCF-7 and ZR-75-1 cells and their anthracycline-, and taxane-resistant derivatives, were characterized through multi-omics, Seahorse-based metabolic flux, and pharmacological inhibition analyses, alongside validation in patient transcriptomic datasets.

**Results:** Chemoresistant cells displayed reduced drug sensitivity and improved recovery following treatment withdrawal. Transcriptomic profiling revealed extensive yet largely distinct gene expression changes among resistant models, whereas metabolomics showed limited divergence. Functional studies demonstrated concurrent reductions in mitochondrial respiration and glycolytic capacity, indicating a low-bioenergetic phenotype without compensatory metabolic rewiring. Lipidomic changes were heterogeneous and model-dependent. Despite this overall metabolic constraint, spermidine and spermine levels were increased across all resistant models, whereas upregulation of the polyamine-related genes *ATP13A4* and *SAT1* was specific to anthracycline-resistant cells. Chemoresistant phenotypes conferred reduced sensitivity to mitochondrial inhibitors. In contrast, sensitivity to polyamine pathway inhibition varied across models. Clinical datasets corroborated key experimental features, showing increased ATP binding cassette subfamily B member 1 (*ABCB1*) expression and significant post-treatment suppression of oxidative phosphorylation and glycolysis signatures. Polyamine pathway enrichment trends were less uniform, reflecting clinical heterogeneity.

**Conclusion:** Chemoresistance in HR+/HER2- BC is characterized by heterogeneous transcriptional remodeling but relative metabolic constraint. Clinical dataset validation confirms post-chemotherapy bioenergetic suppression, while polyamine alterations represent context-dependent adaptations rather than universal vulnerabilities.

## INTRODUCTION

Breast cancer (BC) stands as the leading cause of cancer-related deaths and the most common malignant diagnosis in women worldwide^[1]^. Among its established molecular subtypes, hormone receptor-positive/human epidermal growth factor receptor 2-negative (HR+/HER2-) tumors are the most prevalent, accounting for nearly 75% of the total clinical burden. While the majority of patients with HR+/HER2- disease are diagnosed early and initially present with favorable outcomes, individuals with high-risk clinical features, such as large primary lesions or nodal involvement, require aggressive therapeutic intervention to prevent disease recurrence^[2]^. For this high-risk population, neoadjuvant chemotherapy (NAC) featuring sequential anthracycline and taxane regimens remains a cornerstone of treatment^[2]^. This pre-operative strategy not only facilitates tumor downstaging to improve breast-conserving surgery rates, but also offers a valuable window to monitor real-time, *in vivo* tumor sensitivity to cytotoxic agents^[3]^. However, HR+/HER2- BC displays the lowest pathological complete response rates among all BC subtypes^[4]^. Indeed, approximately 80% of patients harbor substantial residual disease at surgery, and about 40% of these patients ultimately develop metastatic relapse^[5]^. These clinical observations indicate that, despite an initial reduction in tumor burden, a fraction of cancer cells survives anthracycline- and/or taxane-based chemotherapy and persists as a reservoir for disease recurrence.

Resistance to NAC in HR+/HER2- BC is increasingly recognized as a consequence of tumor cell plasticity rather than fixed genetic alterations. Large-scale genomic analyses comparing tumors before and after chemotherapy have failed to identify recurrent mutations that consistently account for treatment failure^[6-11]^. Instead, resistance is thought to arise predominantly through non-genetic, adaptive mechanisms, including the acquisition of transient drug-tolerant states that allow cancer cells to survive chemotherapy-induced stress and later re-enter proliferative programs^[12]^.

Metabolic reprogramming represents a central component of such adaptive responses^[13]^. Cytotoxic agents such as anthracyclines and taxanes impose severe bioenergetic and biosynthetic constraints, challenging cancer cell survival and necessitating rapid adjustments in metabolic pathways. While chemotherapy-associated metabolic adaptations have been reported in triple-negative and HER2-amplified BC^[14-18]^, the metabolic basis of acquired resistance in HR+/HER2- disease remains largely unexplored, particularly in response to distinct drug classes.

Here, we aimed to define the metabolic adaptations associated with resistance to anthracyclines and taxanes in HR+/HER2- BC. Specifically, we investigated whether resistance converges toward shared metabolic programs or relies on drug-specific adaptations, with the goal of identifying actionable metabolic vulnerabilities.

## METHODS

### Cell culture

Human HR+/HER2- BC cell lines used in this study included parental ZR-75-1 cells (ATCC cat. #CRL-1500; RRID:CVCL_0588), paclitaxel-resistant ZR-75-1 (Pac-R), epirubicin-resistant ZR-75-1 (Epi-R), parental MCF-7 cells (ATCC cat. #HTB-22; RRID:CVCL_0031), epirubicin-resistant MCF-7 (Epi-R), and docetaxel-resistant MCF-7 (Doc-R). Parental lines obtained from the American Type Culture Collection (ATCC) were authenticated by the supplier via short tandem repeat (STR) profiling prior to distribution. Parental ZR-75-1 cells and their Pac-R and Epi-R derivatives, as well as parental MCF-7 cells and their Epi-R derivatives, were kindly provided by Dr. Melanie Spears (Ontario Institute for Cancer Research, Toronto, ON, Canada). MCF-7 Doc-R cells were kindly provided by Prof. Martin Michaelis (School of Biosciences, University of Kent, Canterbury, United Kingdom). ZR-75-1 resistant derivatives were generated as previously described^[19]^. The docetaxel-resistant MCF-7 model was developed through sustained exposure to incrementally increasing doses of docetaxel, according to a previously described protocol^[20]^. Similarly, epirubicin-resistant sublines were established by subjecting parental populations to stepwise escalations of epirubicin, initiated at a starting concentration of 0.5 nM. Chemoresistant phenotypes were confirmed when the half-maximal inhibitory concentration (IC_50_) significantly surpassed that of the matching parental cell line, and the cultures reached a selection plateau where they could no longer tolerate higher drug pressures^[21]^. While formal RRIDs are unavailable for these secondary resistant derivatives, their identities and resistance phenotypes were functionally validated through continuous selective pressure and routine dose-response viability assays.

All cell lines were cultured in Dulbecco’s Modified Eagle Medium (DMEM), low glucose, supplemented with GlutaMAX™ and sodium pyruvate (#10567014, Gibco), 10% heat-inactivated fetal bovine serum (FBS; #F7524, Merck Life Science), and 1% penicillin-streptomycin (#15140‐122, Gibco). Cells were maintained at 37 °C in a humidified atmosphere containing 5% CO_2_. In the current study, chemoresistant cell lines were continuously cultured in the presence of chemotherapeutic agents as follows: 25 nM paclitaxel for ZR-75-1 Pac-R and MCF-7 Doc-R cells, 10 nM epirubicin for ZR-75-1 Epi-R cells, and 30 nM epirubicin for MCF-7 Epi-R cells. MCF-7 Doc-R cells were originally established and maintained by the providing laboratory in 20 ng/mL docetaxel; upon receipt, cultures were routinely maintained in our laboratory under continuous exposure to 25 nM paclitaxel, which shares the same taxane class and microtubule-stabilizing mechanism as docetaxel. Paclitaxel (6 mg/mL; Fresenius Kabi, lot no. 87230210AB) and epirubicin (Farmorubicin, 2 mg/mL; Pfizer, batch no. FR32501) were obtained as clinical-grade formulations from the hospital pharmacy of Cliniques universitaires Saint-Luc (Brussels, Belgium) and diluted in culture medium immediately prior to use. Cell lines were systematically screened for mycoplasma contamination utilizing a polymerase chain reaction (PCR)-based detection platform (MycoplasmaCheck, Eurofins Genomics), confirmed to be strictly mycoplasma-negative prior to use, and maintained below 20 passages post-thawing to prevent phenotypic drift.

### Cell viability assays

To assess the effects of chemotherapeutic agents on cell viability and growth capacity over time, cells were seeded in triplicate in 6-well plates at a density of 2.5 × 10^5^ cells/mL for ZR-75-1 cells and 2.0 × 10^5^ cells/mL for MCF-7 cells, and allowed to adhere for 24 h. Baseline viability was measured using the PrestoBlue™ cell viability reagent (#A13262, Thermo Fisher Scientific) according to the manufacturer’s instructions. Cells were then treated with paclitaxel (25 nM) or epirubicin (10 nM for ZR-75-1 cells; 30 nM for MCF-7 cells) for 72 h, after which cell viability was reassessed. Following drug exposure, cells were washed and cultured in drug-free medium. Long-term proliferative recovery was evaluated at 4 and 7 days post-washout using PrestoBlue™. Cell growth kinetics were independently assessed by direct cell counting. Cells were seeded in triplicate in 24-well plates (50,000 cells per well), and viable cell numbers were determined on days 1, 2, 3, 4, and 7 using Trypan Blue exclusion and a hemocytometer.

To evaluate sensitivity to metabolic inhibitors, ZR-75-1 cells (1.5 × 10^4^ cells/well) and MCF-7 cells (7.5 × 10^3^ cells/well) were seeded in 96-well plates (≥ 3 technical replicates per condition) and allowed to adhere for 24 h. Cells were then treated with antimycin A (#33357, Cell Signaling Technology), IACS-010759 (#HY-112037, MedChemExpress), mitoguazone [methylglyoxal-bis(guanylhydrazone); MGBG] (#HY-106634, MedChemExpress), difluoromethylornithine (DFMO) hydrochloride hydrate (#HY-B0744B, MedChemExpress) or AMXT-1501 tetrahydrochloride (HY-124617A, MedChemExpress) for 72 h at different concentrations, as indicated in the figure legends. Cell viability was subsequently assessed using the PrestoBlue™ assay according to the manufacturer’s instructions. All cell viability and cell counting experiments included three technical replicate wells per condition and were performed across three independent biological experiments (*n* = 3). Technical replicates within each run were averaged to yield a single value per biological replicate; thus, every individual data point depicted in the corresponding figures represents an independent biological replicate.

### Seahorse analysis

Real-time monitoring of oxygen consumption rates (OCRs) and extracellular acidification rates (ECARs) was performed using a Seahorse XFe96 Extracellular Flux Analyzer (Agilent Technologies). Cells (1 × 10^4^ cells/well) were seeded in specialized XF96 cell culture microplates and allowed to fully adhere overnight. Prior to initiating the assay, the growth medium was replaced with specialized XF assay medium containing 2 mM L-glutamine (and 10 mM glucose for XF Cell MitoStress Test), adjusted to pH 7.4, followed by a 1-hour equilibration period at 37 °C in a non-CO_2_ incubator. Bioenergetic profiles were mapped via sequential automatic injections of metabolic inhibitors: 1 μM oligomycin, 2 μM carbonyl cyanide-4 (trifluoromethoxy)phenylhydrazone (FCCP) and 0.5 μM rotenone/antimycin A to assess mitochondrial function parameters; 10 mM glucose, 1 µM oligomycin and 50 mM 2-deoxyglucose (2-DG) to evaluate glycolysis activity. Specifically, glucose-dependent ECAR was calculated by comparing the values before and after the addition of the substrate. All measurements were normalized to total protein content per well and expressed in mpH/min/μg protein (ECAR) or pmoles/min/μg protein (OCR).

### Dosage of extracellular glucose and lactate

Cellular consumption of glucose and secretion of lactate were monitored by seeding cells at 3 × 10^5^ cells/well in 12-well plates utilizing 500 µL of routine culture media. After 24 h, monolayers were washed and replenished with 500 µL of DMEM containing 10 mM D-glucose (#G8270, Sigma-Aldrich), 2 mM L-glutamine and 10% dialyzed FBS (#F0392, Sigma-Aldrich). Baseline metabolite profiles were verified using incubated, cell-free medium controls. Following a 24-hour treatment period, the extracellular media were harvested, transferred to 10 kDa centrifugal filter tubes (VWR), and spun at 10,000 × *g* (15 min, 4 °C) for deproteinization. Quantitative profiling of glucose and lactate in the prepared filtrates (50 µL sample volume) was performed on an ISCUSflex automated microdialysis analyzer (Aurora Borealis) using specific enzymatic reagents (CMA Microdialysis AB). Net metabolic fluxes were computed relative to the cell-free blank controls, normalized against total cellular protein per well, and reported in units of µmol/hr/mg protein.

### Quantitative real-time PCR and high-throughput RNA sequencing

Isolation of total RNA was performed using the Monarch Total RNA Miniprep Kit (New England Biolabs) in strict accordance with the manufacturer’s protocol. The quantitative yield and chemical purity of the isolated RNA specimens were determined by spectrophotometry using a NanoDrop 1000 (Thermo Fisher Scientific).

For RNA sequencing, three biological replicates (*n* = 3) per experimental group, derived from independent cell cultures and separate RNA extractions, were processed. Ribosomal RNA (rRNA) depletion was performed using the RiboCop rRNA Depletion Kit (Lexogen). RNA-seq libraries were subsequently prepared from rRNA-depleted RNA using the NEBNext Ultra II Directional RNA Library Prep Kit for Illumina (New England Biolabs). Libraries were paired-end sequenced (2 × 100 base pairs) on an Illumina NextSeq2000 platform, achieving an average sequencing depth of approximately 20 million reads per sample. For quantitative real-time PCR, total RNA (1 µg) was reverse transcribed with the RevertAid Reverse-Transcriptase, oligo-dT and random hexamers (Thermo Fisher Scientific) before quantitative PCR amplification on a ViiA 7 real-time PCR system (Applied Biosystems) using SYBR® select master mix for cfx (Thermo Fisher Scientific) and gene-specific primer sequences [Supplementary Table 1]. Data were analyzed according to ddCt method using human *GTF2B* as reference gene.

### Metabolomics analysis

Untargeted metabolomics profiling was performed using liquid chromatography-mass spectrometry (LC-MS) (see Supplementary Methods for full details). Briefly, intracellular metabolites were extracted from cell pellets using a methanol/water-based extraction buffer containing N-ethylmaleimide and isotopically labeled internal standards. Chromatographic separation was performed using hydrophilic interaction LC coupled to high-resolution MS operated in both positive and negative electrospray ionization modes. Data acquisition included full-scan MS and data-dependent MS/MS. Raw data were processed using MS-DIAL (version 4.9.221218)^[22]^, including peak detection, alignment, normalization, and metabolite annotation using an in-house spectral library of authentic standards. Metabolite intensities were normalized using both cell numbers and isotopically labeled internal standards. Downstream statistical analyses and visualization were performed using R and Python.

### RNA-seq and metabolomic data processing and statistical analysis

Raw transcriptomic reads underwent initial quality control evaluation using FastQC (Babraham Bioinformatics). Adapter sequences and low-quality bases were trimmed using Trimmomatic. High-quality trimmed reads were then quantified at the transcript level using Salmon (v1.10.3)^[23]^ by mapping to the Ensembl human reference transcriptome (GRCh38, file: *Homo_sapiens.GRCh38.cdna.all.fa*). Differential gene expression analysis was performed using the edgeR Bioconductor package (v.4.8.2)^[24]^, adjusting for multiple testing using the Benjamini-Hochberg false discovery rate (FDR) procedure. Raw metabolomic concentrations were log2-transformed to stabilize variance and approximate a normal distribution. For heatmap visualization, metabolite intensities were separately log10-transformed. Systematic technical variation between samples was corrected by median-centering normalization using the normalizeIntensity function from the qmtools Bioconductor package (v.1.14.0). Differentially abundant metabolites between experimental conditions were identified using linear modeling with empirical Bayes moderation implemented in the limma Bioconductor package (v.3.66.0)^[25]^. To assess the clinical relevance of our findings, we analyzed two publicly available RNA-sequencing datasets of HR+/HER2- BC patients sampled before and after NAC (GSE191127^[26]^ and GSE123845^[27]^). GSE191127 comprises paired pre-treatment biopsies and post-treatment residual tumors (*n* = 16 pairs), whereas GSE123845 includes 43 pre-treatment and 7 post-treatment samples. Differential gene expression between pre- and post-treatment tumors was analyzed in R (v4.4.1) using the lmerSeq package (v0.1.7). Statistical significance was assessed using moderated *t*-tests, followed by Benjamini-Hochberg FDR correction. Gene set enrichment analysis of transcriptomic and metabolomic alterations was subsequently performed against the KEGG and Reactome pathway databases using the fgsea package (v1.36.0). All transcriptomic and metabolomic visualizations, including differential expression and pathway enrichment plots, were generated in R using the ggplot2 ecosystem.

### Lipidomics analysis

Lipidomic profiling was performed using LC-MS, with full methodological details provided in the Supplementary Methods. Briefly, cellular lipids were extracted using a methanol/isopropanol/methyl tert-butyl ether-based protocol in the presence of internal standards. Lipid species were separated by reverse-phase ultra-high-performance LC and analyzed by high-resolution MS operating in both positive and negative ionization modes. Peak integration, feature alignment, and lipid annotation were performed using MS-DIAL (v4.9.221218) and validated against an in-house lipid spectral library. Lipid features were normalized using the B-MIS approach, followed by normalization to cell number and internal standards. Normalized lipid intensities were then log2-transformed prior to statistical analysis and visualization. For heatmap visualization, normalized lipid intensities were separately log10-transformed. Outlier replicates were identified using a multivariate distance-based approach, based on the Euclidean distance of each sample from the group-specific median. Samples displaying substantially increased distances were excluded as outliers. This classification was further supported by principal component analysis, in which outliers separated clearly from the main sample cluster. Based on this quality-control assessment, five biological replicates were excluded from the lipidomic analysis: MCF-7 Parental replicate 1, MCF-7 Doc-R replicate 2, ZR-75-1 Parental replicate 3, ZR-75-1 Pac-R replicate 4, and ZR-75-1 Epi-R replicate 4. Accordingly, three biological replicates were included in the final analysis for each of these groups, while all four replicates were included for MCF-7 Epi-R. Differences in lipid abundance across experimental conditions were assessed using empirical Bayes linear modeling implemented in the limma Bioconductor package (v3.66.0). Log_2_ fold-change values were derived from fitted model coefficients, and *P*-values were calculated from moderated *t*-statistics. In line with the exploratory nature of the analysis, lipid features were prioritized based on raw *P*-values combined with an effect-size threshold of log_2_FC > 0.5. All data visualizations were generated in R using the ggplot2 package.

### Western blot analysis

Subconfluent cell monolayers were rinsed twice with ice-cold phosphate-buffered saline and harvested in radioimmunoprecipitation assay (RIPA) lysis buffer containing protease (Sigma-Aldrich) and phosphatase (Roche) inhibitor cocktails. The resulting lysates were cleared via centrifugation (10,000 × *g*, 10 min, 4 °C) and preserved at -80 °C. Total protein concentrations were quantified using a bicinchoninic acid assay (Thermo Fisher Scientific). Protein samples were mixed with Laemmli buffer containing 100 mM dithiothreitol, denatured at 95 °C for 5 min, resolved by SDS-PAGE on 8%-15% polyacrylamide gels, and electrotransferred onto PVDF membranes. Nonspecific binding sites were blocked using 5% non-fat dry milk (#T145.3, Roth) prepared in TBS-0.1% Tween 20 (TBST). Membranes were then probed overnight at 4 °C with primary antibodies targeting human β-actin (clone AC-15, #A5441, Sigma-Aldrich; 1:10,000) or ATP binding cassette subfamily B member 1 (ABCB1; clone E1Y7S, #13978S, Cell Signaling Technology; 1:1,000). Following washing in TBST, membranes were exposed to horseradish peroxidase-conjugated secondary antibodies (#115-035-003 and #111-035-003, Jackson ImmunoResearch; 1:5,000) for 1 h at room temperature. Protein bands were developed using the ECL Western Blotting Detection Kit (GE Healthcare) and captured on an Amersham Imager 600 system (GE Healthcare). Western blot analyses were performed using three independent biological replicates for each parental and chemoresistant cell line (*n* = 3), designated as samples #1, #2, and #3.

### Statistical analysis and reproducibility

Graphical representations and statistical analyses were performed using GraphPad Prism (version 10.6.0). Data were analyzed using an unpaired Student’s *t*-test, one-way analysis of variance (ANOVA) followed by Dunnett’s multiple-comparison post-hoc test, or two-way ANOVA followed by Tukey’s or Sidák’s multiple-comparison post-hoc tests, as appropriate. Statistical significance was defined as ^*^P < 0.05, ^**^P < 0.01, ^***^P < 0.001. No statistical methods were used to predetermine sample sizes, which were instead selected based on established standards in the field. No samples or individual data points were excluded from the analyses, except for five biological replicates excluded from the lipidomic analysis following the quality-control procedure described in the Lipidomics Analysis section.

## RESULTS

### Chemoresistant HR+/HER2- BC models display stable and functionally validated resistance phenotypes

**Figure 1:**
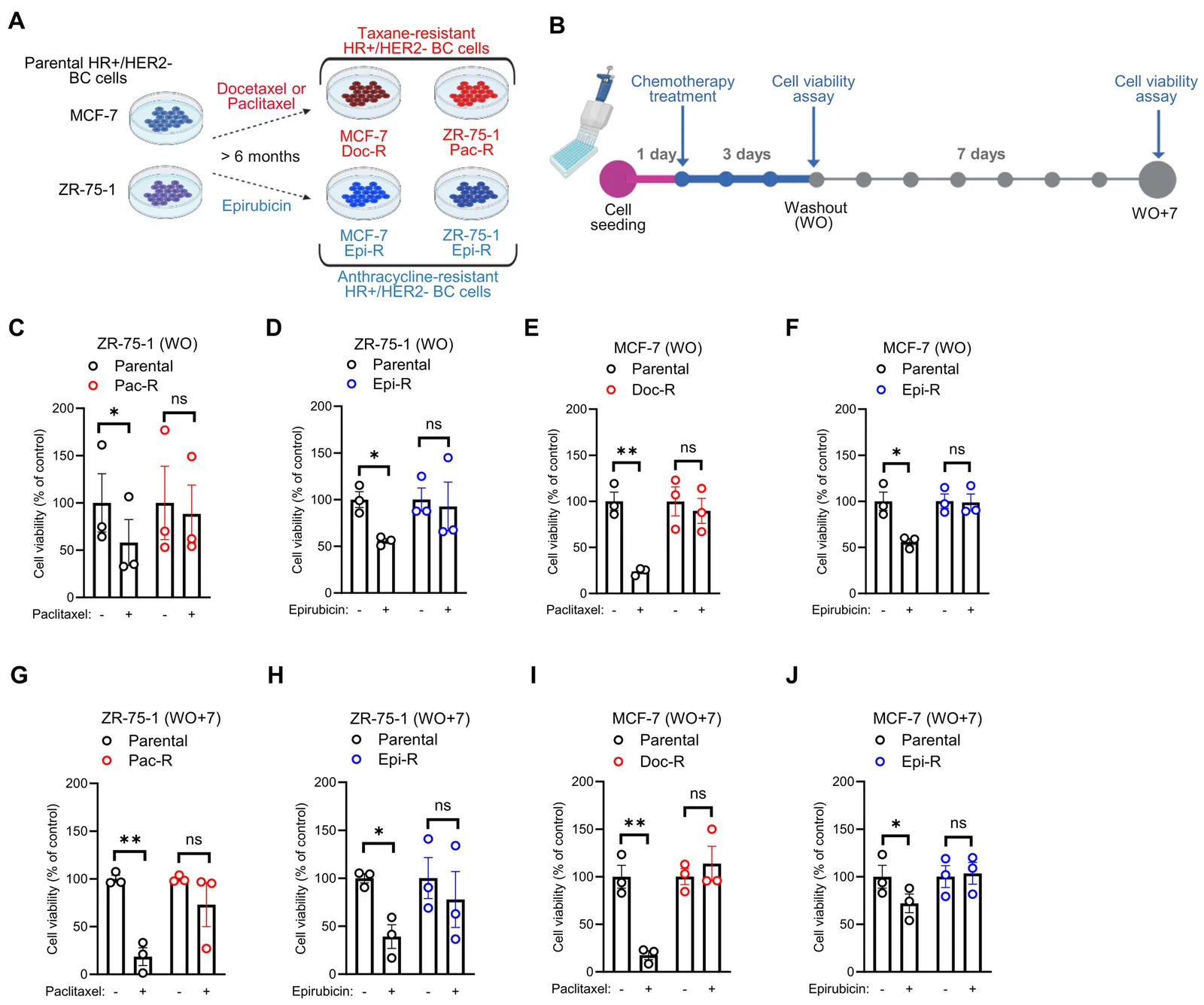
Drug response profiling validates stable chemoresistant phenotypes in HR+/HER2- BC models. (A) Schematic representation of the generation of anthracycline- and taxane-resistant cell populations from parental HR+/HER2- BC cell lines (MCF-7 and ZR-75-1). Created in BioRender. Corbet, C. (2026) https://BioRender.com/i5zffea; (B) Experimental design of the drug challenge and recovery assays. Cells were treated with chemotherapeutic agents for 72 h, followed by drug WO and a 7-day recovery period (WO+7). Created in BioRender. Corbet, C. (2026) https://BioRender.com/eoukvmv; (C and D) Viability of parental and drug-resistant ZR-75-1 cells after 72 h of treatment (WO) with 25 nM paclitaxel (C) or 10 nM epirubicin (D); (E and F) Viability of parental and drug-resistant MCF-7 cells after 72 h of treatment (WO) with 25 nM paclitaxel (E) or 30 nM epirubicin (F); (G and H) Viability of parental and drug-resistant ZR-75-1 cells following 72 h of treatment with 25 nM paclitaxel (G) or 10 nM epirubicin (H), and subsequent culture in drug-free medium for 7 days (WO+7); (I and J) Viability of parental and drug-resistant MCF-7 cells following 72 h of treatment with 25 nM paclitaxel (I) or 30 nM epirubicin (J), and subsequent culture in drug-free medium for 7 days (WO+7). Data are presented as mean ± SEM from three independent biological experiments (*n* = 3), each performed with three technical replicate wells per condition (C-J). Individual data points represent the mean of the technical replicates from each independent biological experiment. Statistical significance was determined using two-way ANOVA with Sidák’s multiple comparison test (C-J). ^*^P < 0.05; ^**^P < 0.01; ns: not significant. HR+/HER2-: Hormone receptor-positive/human epidermal growth factor receptor 2-negative; BC: breast cancer; WO: washout; SEM: standard error of the mean; ANOVA: analysis of variance; Doc-R: docetaxel-resistant; Pac-R: paclitaxel-resistant; Epi-R: epirubicin-resistant.

To investigate metabolic alterations associated with acquired resistance to anthracyclines and taxanes, we employed a panel of parental and chemoresistant HR+/HER2- BC cell models derived from ZR-75-1 and MCF-7 cell lines [Figure 1A]. Paclitaxel-resistant ZR-75-1 cells (Pac-R) have previously been shown to exhibit a marked reduction in paclitaxel sensitivity, with an approximately 170-fold increase in IC_50_ compared to parental cells, as well as cross-resistance to other taxanes^[19]^. However, the additional chemoresistant derivatives used in the present study, including epirubicin-resistant ZR-75-1 and MCF-7 (Epi-R), and docetaxel-resistant MCF-7 cells (Doc-R), had not been systematically evaluated under the treatment and recovery conditions required for downstream multi-omics analyses. We therefore implemented a standardized drug challenge and recovery assay to validate the functional stability of all resistant cell populations [Figure 1B]. Cells were exposed to paclitaxel or epirubicin at concentrations corresponding to their respective resistance selection, and responses were assessed both at the end of treatment and following a defined drug-free recovery period.

Across both ZR-75-1 and MCF-7 models, chemoresistant derivatives consistently displayed reduced drug sensitivity and enhanced capacity to resume growth following drug withdrawal compared with parental cells [Figure 1C-J and Supplementary Figure 1A-F]. These results indicate that the selected models represent stable chemoresistant phenotypes rather than transient drug-tolerant states, supporting their use for subsequent systems-level analyses.

### Transcriptomic analyses reveal extensive but largely non-overlapping gene expression reprogramming in chemoresistant cells

**Figure 2:**
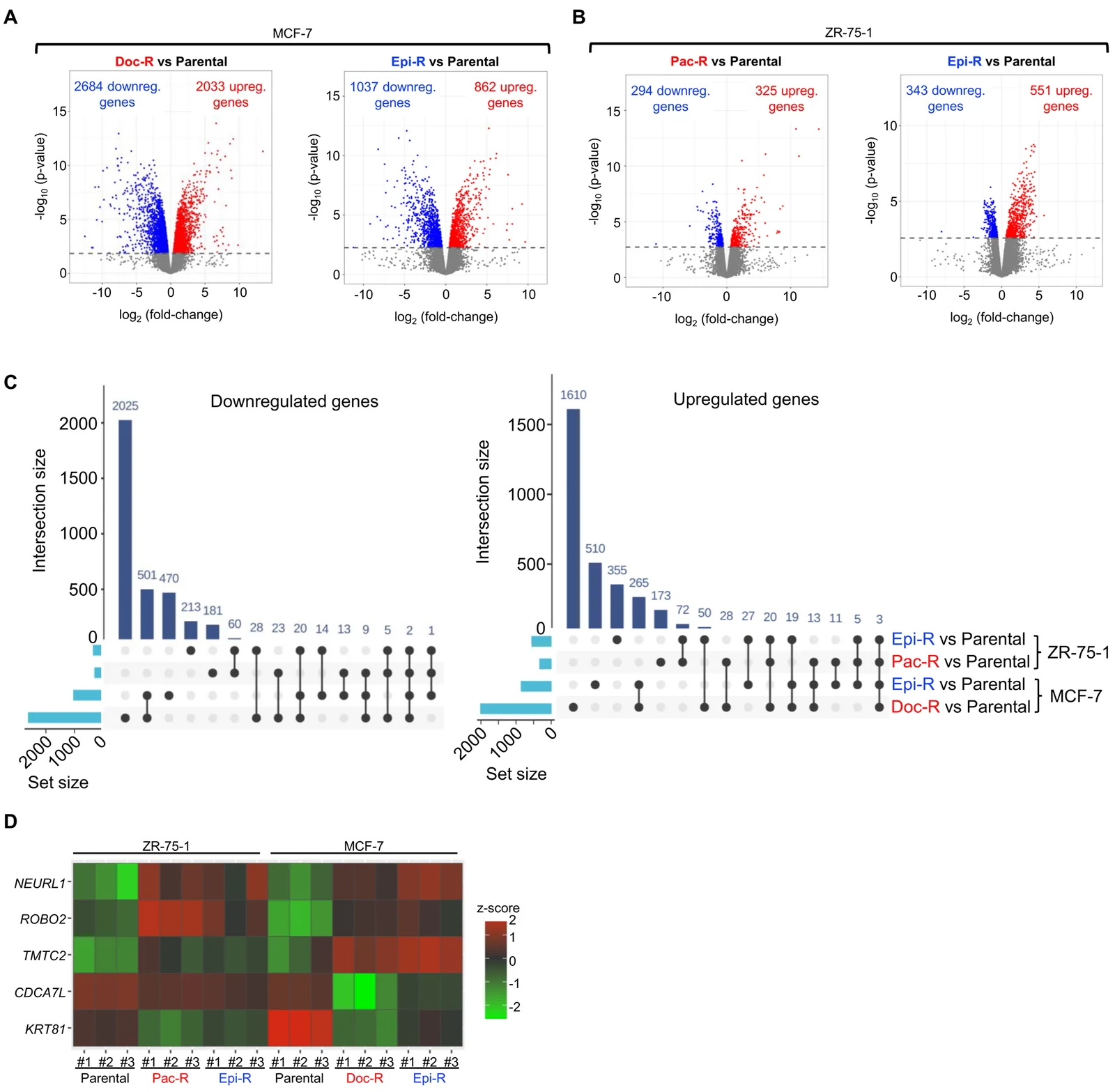
Transcriptomic profiling reveals extensive but heterogeneous gene expression remodeling in chemoresistant HR+/HER2- BC cells. (A and B) Volcano plots depicting differentially expressed genes in chemoresistant MCF-7 cells (A) and ZR-75-1 (B) cells compared with their respective parental counterparts. Differentially expressed genes were identified using a threshold of FDR < 0.05 without imposing an absolute log_2_FC cutoff, and were classified as upregulated (log_2_FC > 0) or downregulated (log_2_FC < 0) according to the direction of change; (C) UpSet plots illustrating the overlap of differentially expressed genes (logFC < -0.5 or > 0.5; FDR < 0.05) across all chemoresistant models; (D) Heatmap showing genes consistently dysregulated (logFC < -0.5 or > 0.5; FDR < 0.05) across taxane- and anthracycline-resistant HR+/HER2- BC cell models. HR+/HER2-: Hormone receptor-positive/human epidermal growth factor receptor 2-negative; BC: breast cancer; FDR: false discovery rate; Doc-R: docetaxel-resistant; Epi-R: epirubicin-resistant; Pac-R: paclitaxel-resistant.

To characterize molecular alterations associated with acquired resistance to anthracyclines and taxanes, we performed RNA sequencing in parental and chemoresistant HR+/HER2- BC cells. Principal component analysis revealed a clear separation between parental and resistant populations in both MCF-7 and ZR-75-1 models, indicating substantial transcriptional reprogramming upon acquisition of drug resistance [Supplementary Figure 2A and B]. Differential gene expression analysis confirmed widespread transcriptomic remodeling across all resistant models [Figure 2A and B], although the magnitude of these changes varied, with MCF-7 Doc-R cells displaying the largest number of dysregulated genes. Despite this extensive reprogramming, overlap in differentially expressed genes across all resistant models remained limited [Figure 2C and D], indicating that resistance is predominantly driven by context-dependent transcriptional adaptations rather than a shared gene expression program. Consistently, only minimal overlap was observed between MCF-7 and ZR-75-1 cells resistant to the same class of chemotherapeutic agents [Supplementary Figure 2C], whereas greater similarity was observed within models derived from the same parental cell line [Supplementary Figure 2D].

**Figure 3:**
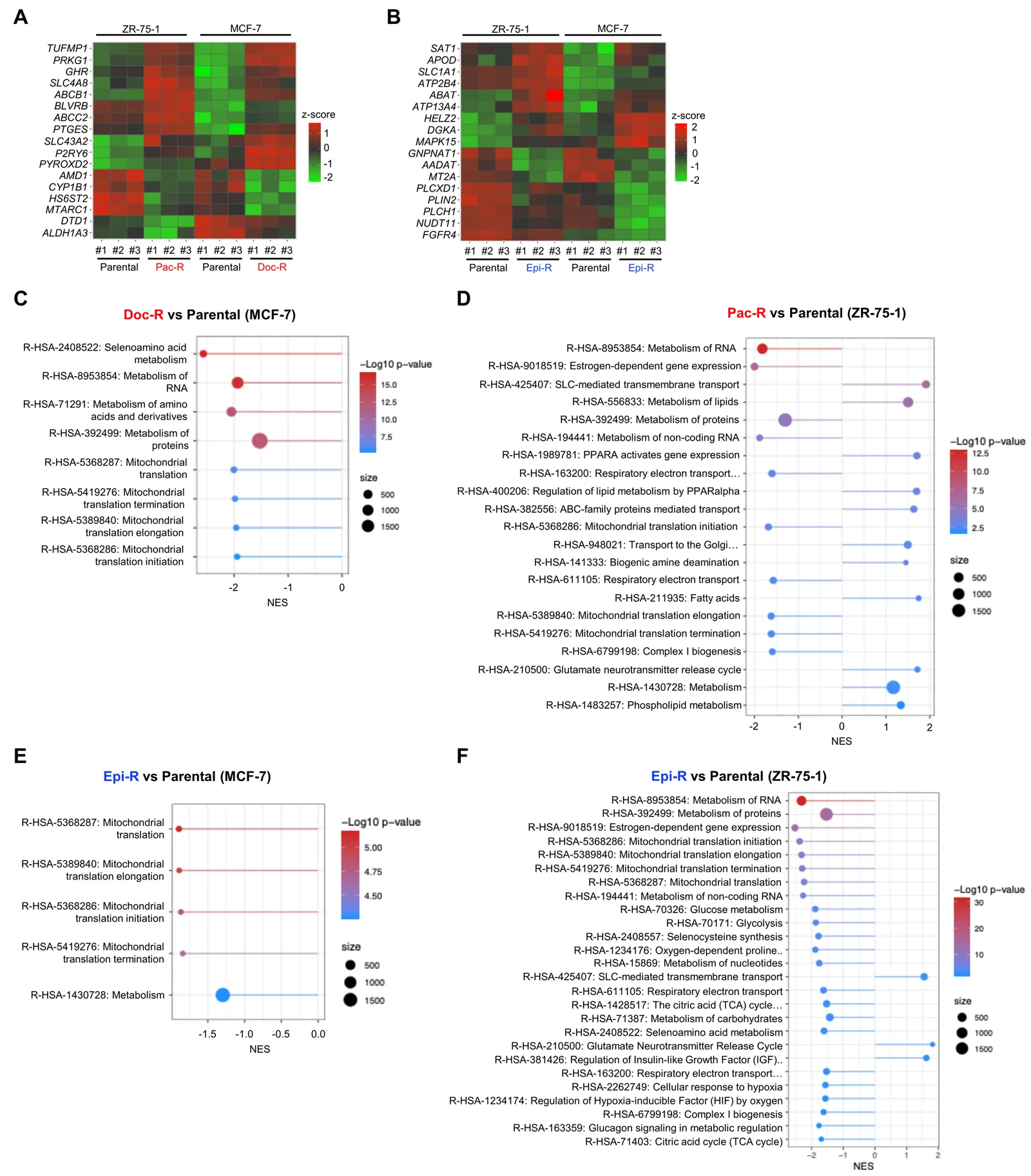
Drug-specific transcriptional reprogramming of metabolism pathways in chemoresistant HR+/HER2- BC. (A and B) Heatmaps of metabolism-related genes consistently dysregulated (logFC < -0.5 or > 0.5; FDR < 0.05) in taxane-resistant (A) and anthracycline-resistant (B) cell models; (C-F) Pathway enrichment analysis highlighting dysregulated metabolic pathways in MCF-7 Doc-R (C), ZR-75-1 Pac-R (D), MCF-7 Epi-R (E), and ZR-75-1 Epi-R (F) cells compared with their respective parental counterparts. HR+/HER2-: Hormone receptor-positive/human epidermal growth factor receptor 2-negative; BC: breast cancer; FDR: false discovery rate; Doc-R: docetaxel-resistant; Pac-R: paclitaxel-resistant; Epi-R: epirubicin-resistant; NES: normalized enrichment score.

Functional annotation revealed partially convergent yet drug-biased transcriptional programs. Taxane-resistant models were characterized by the upregulation of genes involved in drug efflux (e.g., *ABCB1*, *ABCC2*) and redox homeostasis (e.g., *ALDH1A3*, *BLVRB*, and *CYP1B1*) [Figure 3A and B]. The preferential upregulation of ABCB1 in taxane-resistant cells was further validated by qRT-PCR in both models and corroborated at the protein level by Western blot analysis [Supplementary Figure 3A-D]. In contrast, anthracycline-resistant cells preferentially exhibited alterations in lipid metabolism-related genes (e.g., *APOD*, *DGKA*, and *PLIN2*) alongside increased expression of *ATP13A4* and spermidine/spermine N1-acetyltransferase 1 (*SAT1*), two genes implicated in polyamine transport and catabolism^[28]^. qRT-PCR analysis confirmed increased *SAT1* expression in anthracycline-resistant MCF-7 and ZR-75-1 cells, whereas *ATP13A4* expression was significantly increased only in MCF-7 Epi-R cells, with a non-significant upward trend in ZR-75-1 Epi-R cells [Supplementary Figure 3E-H]. Pathway enrichment analysis further highlighted the marked heterogeneity of resistance-associated transcriptional changes. Mitochondrial translation emerged as the only pathway consistently downregulated across all chemoresistant models, irrespective of the drug used [Figure 3C-F]. Beyond this shared feature, most pathways were regulated in a cell line- and drug-dependent manner. Notably, pathways related to mitochondrial function, glucose metabolism, lipid metabolism, and polyamine homeostasis were recurrently affected suggesting potential metabolic adaptations associated with chemoresistance. However, whether these transcriptional changes translate into functional metabolic rewiring remained unclear.

### Chemoresistant HR+/HER2- BC cells exhibit reduced mitochondrial respiration and glycolytic capacity

**Figure 4:**
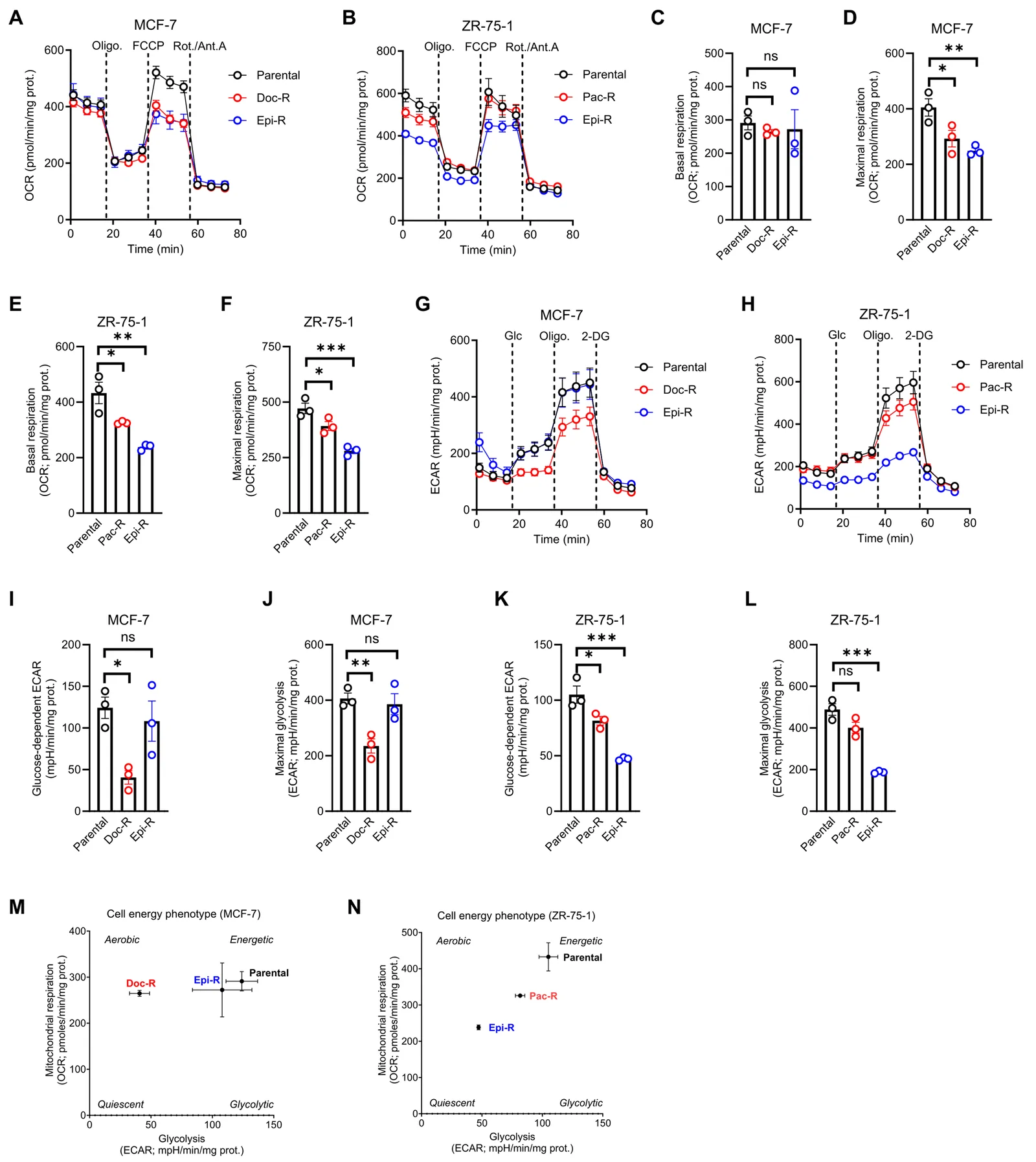
Chemoresistant HR+/HER2- BC cells exhibit reduced mitochondrial respiration and glycolytic capacity. (A and B) OCR profiles in parental and chemoresistant MCF-7 (A) and ZR-75-1 (B) cells following sequential treatment with 1 µM oligomycin, 2 µM FCCP and 0.5 µM rotenone/antimycin A; (C-F) Quantification of basal (C and E) and maximal (D and F) mitochondrial respiration in parental and chemoresistant MCF-7 (C and D) and ZR-75-1 (E and F) cells; (G and H) ECAR profiles in parental and chemoresistant MCF-7 (G) and ZR-75-1 (H) cells following sequential treatment with 10 mM glucose, 1 µM oligomycin and 50 mM 2-DG; (I-L) Quantification of glycolysis (I and K) and maximal glycolytic capacity (J and L) in parental and chemoresistant MCF-7 (I and J) and ZR-75-1 (K and L) cells; (M and N) Seahorse-based energetic maps (i.e., mitochondrial respiration *vs.* glycolysis) in parental and chemoresistant MCF-7 (M) and ZR-75-1 (N) cells after evaluation of basal OCR and ECAR. Data are presented as mean ± SEM from three independent biological experiments (*n* = 3), each performed with five technical replicate wells per condition (A-N). Individual data points represent the mean of the technical replicates from each independent biological experiment. Statistical significance was determined using one-way ANOVA with Dunnett’s multiple comparison test (C-F and I-L). ^*^P < 0.05; ^**^P < 0.01; ^***^P < 0.001; ns: not significant. HR+/HER2-: Hormone receptor-positive/human epidermal growth factor receptor 2-negative; BC: breast cancer; OCR: oxygen consumption rate; FCCP: carbonyl cyanide-4 (trifluoromethoxy)phenylhydrazone; ECAR: extracellular acidification rate; 2-DG: 2-deoxyglucose; SEM: standard error of the mean; ANOVA: analysis of variance; Doc-R: docetaxel-resistant; Epi-R: epirubicin-resistant; Pac-R: paclitaxel-resistant.

To determine whether transcriptional alterations were associated with functional metabolic changes, we assessed cellular bioenergetics using Seahorse extracellular flux analysis. OCR measurements revealed an overall reduction in mitochondrial respiration in chemoresistant cells compared with their parental counterparts [Figure 4A and B]. While basal respiration showed modest changes in MCF-7 models, maximal respiratory capacity was significantly decreased, particularly in Epi-R cells [Figure 4C and D]. In ZR-75-1 cells, both basal and maximal respiration were markedly reduced in Pac-R and Epi-R conditions, indicating a pronounced impairment of mitochondrial function [Figure 4E and F]. These functional alterations are consistent with transcriptomic data identifying mitochondrial translation as the only pathway uniformly downregulated across all chemoresistant models [Figure 3C-F].

ECAR analysis further showed that glycolytic activity was not increased to compensate for reduced mitochondrial function. Instead, glycolysis and maximal glycolytic capacity were decreased, particularly in ZR-75-1 Epi-R cells, with more variable effects observed in MCF-7 models [Figure 4G-L]. Consistently, glucose consumption and lactate secretion were not elevated in chemoresistant cells [Supplementary Figure 4A-D].

Together, these data indicate that chemoresistant cells adopt a low-bioenergetic phenotype characterized by coordinated reductions in both mitochondrial respiration and glycolytic activity, suggesting a global suppression or reprogramming of energy metabolism as illustrated in cell energy maps [Figure 4M and N].

### Metabolomic profiling reveals limited global divergence but highlights polyamine metabolism as a recurrent feature of chemoresistance

**Figure 5:**
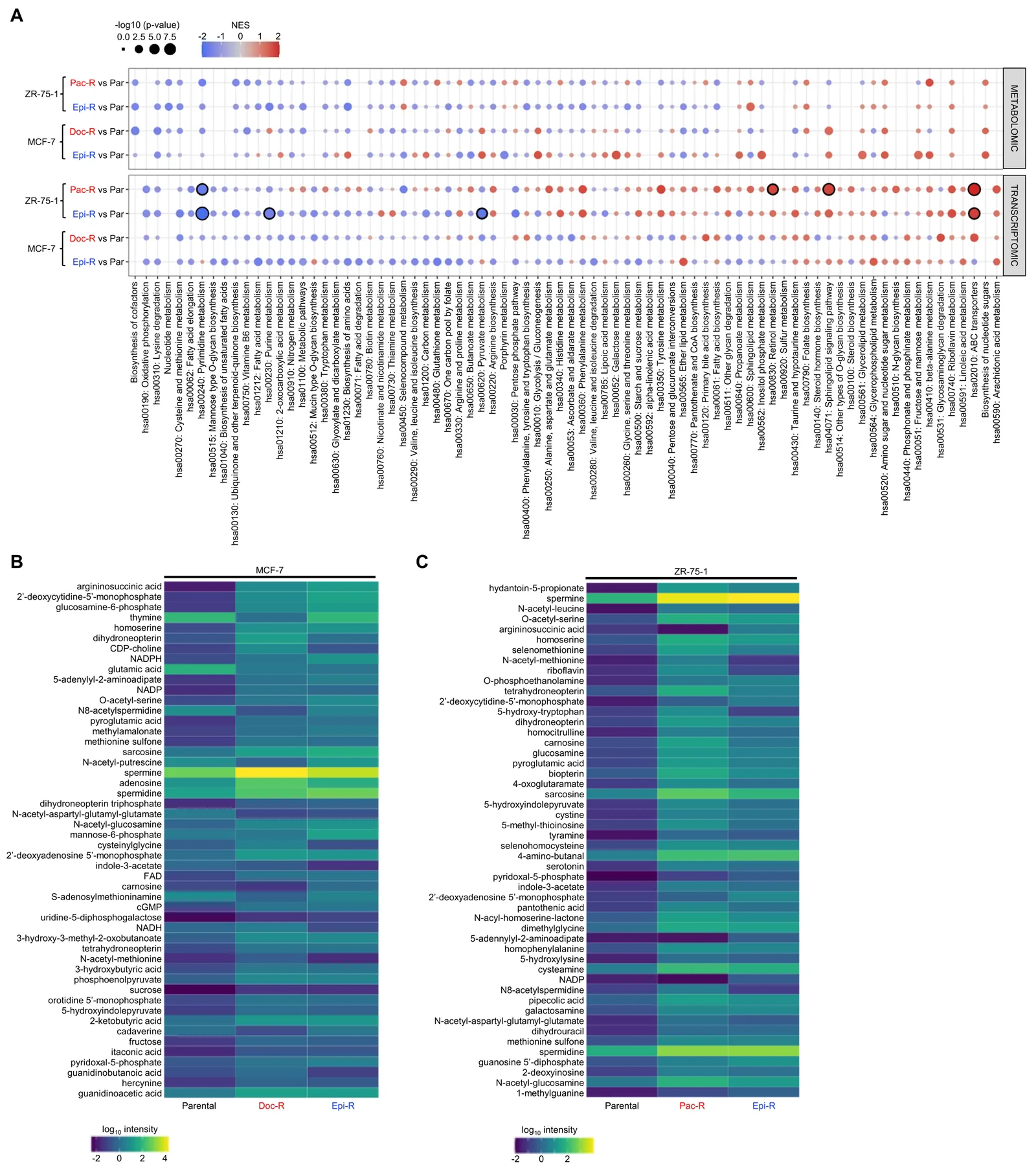
Metabolomic profiling reveals limited global divergence but identifies selective metabolic alterations in chemoresistant HR+/HER2- BC cells. (A) Integrated transcriptomic and metabolomic pathway enrichment analysis highlighting dysregulated metabolic pathways in taxane- and anthracycline-resistant models; (B and C) Heatmaps of the top 50 most regulated metabolites in chemoresistant MCF-7 (B) and ZR-75-1 (C) cell models relative to their parental counterparts. Color scale represents log10-transformed intensities. HR+/HER2-: Hormone receptor-positive/human epidermal growth factor receptor 2-negative; BC: breast cancer; NES: normalized enrichment score; Pac-R: paclitaxel-resistant; Epi-R: epirubicin-resistant; Doc-R: docetaxel-resistant.

To directly assess metabolic alterations, we performed untargeted metabolomic analyses. Principal component analysis revealed substantial overlap between parental and chemoresistant cells in both MCF-7 and ZR-75-1 models [Supplementary Figure 5A and B], indicating that global metabolic profiles remain relatively conserved despite pronounced transcriptional divergence. Consistently, integrated pathway analysis combining transcriptomic and metabolomic data revealed limited concordance across omics layers [Figure 5A]. While purine and pyrimidine metabolism were decreased at both levels, most pathways did not show consistent regulation. Core bioenergetic pathways, including oxidative phosphorylation (OXPHOS) and fatty acid metabolism, displayed an overall decreasing trend, in agreement with functional analyses. At the metabolite level, changes were heterogeneous and not always aligned with pathway-level predictions [Figure 5B and C]. Notably, polyamine metabolism emerged as one of the most recurrently altered features across chemoresistant models. Increased levels of spermidine and spermine were consistently observed, although other intermediates (e.g., N8-acetylspermidine, N-acetylputrescine) displayed context-dependent regulation, indicating partial rather than uniform pathway activation.

### Lipidomic profiling reveals heterogeneous but predominantly reduced lipid states in chemoresistant models

**Figure 6:**
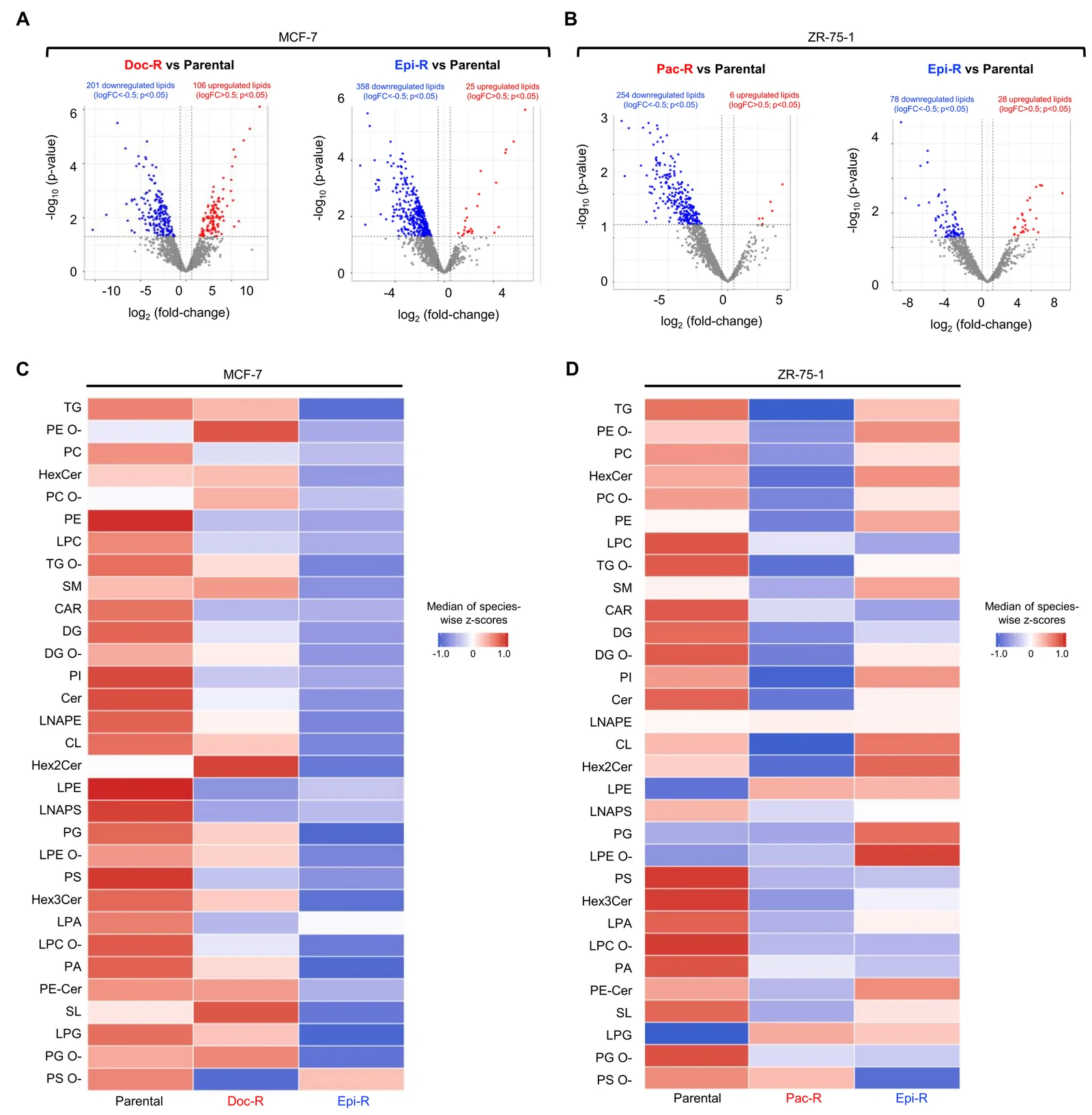
Lipidomic profiling reveals remodeling of lipid species in chemoresistant HR+/HER2- BC cells. (A and B) Volcano plots showing differentially abundant lipid species (logFC < -0.5 or > 0.5; *P* < 0.05) in chemoresistant MCF-7 (A) and ZR-75-1 (B) cells compared with parental cells; (C and D) Heatmaps summarizing lipid class distribution in chemoresistant MCF-7 (C) and ZR-75-1 (D) cells relative to their respective parental counterparts. Values represent median row z-scores of lipid species within each class following log_10_ transformation and averaging across biological replicates. HR+/HER2-: Hormone receptor-positive/human epidermal growth factor receptor 2-negative; BC: breast cancer; Doc-R: docetaxel-resistant; Epi-R: epirubicin-resistant; Pac-R: paclitaxel-resistant.

To assess lipid alterations, we performed untargeted lipidomic analyses across parental and chemoresistant models. Principal component analysis revealed partial separation between parental and resistant cells in both MCF-7 and ZR-75-1 models [Supplementary Figure 6A and B], indicating modest but detectable lipidomic divergence. Volcano plot analysis demonstrated a strong bias toward lipid downregulation in resistant cells, although the extent varied depending on both drug and cell line. In MCF-7 cells, Epi-R cells exhibited a pronounced reduction in lipid species (358 downregulated *vs.* 25 upregulated), whereas Doc-R cells showed a more balanced distribution [Figure 6A]. In contrast, in ZR-75-1 cells, Pac-R cells displayed the most extensive lipid depletion (254 downregulated *vs.* 6 upregulated), while Epi-R cells showed a comparatively milder, yet still reduction profile (78 downregulated *vs.* 28 upregulated) [Figure 6B]. These findings indicate that the magnitude of lipid depletion is both drug- and model-dependent. Overlap between MCF-7 and ZR-75-1 cells resistant to the same class of chemotherapeutic agents remained limited [Supplementary Figure 6C], whereas models originating from the same parental cell line showed greater similarity [Supplementary Figure 6D], consistent with observations at the transcriptomic level. At the lipid class level, lipid abundance was broadly decreased, particularly in MCF-7 Epi-R and ZR-75-1 Pac-R cells. Neutral lipids were consistently reduced across resistant models, suggesting a decrease in lipid storage capacity. Phospholipids and sphingolipids were also predominantly decreased in resistant cells, although selective increases were observed in specific contexts, particularly in MCF-7 Doc-R and ZR-75-1 Epi-R models [Figure 6C and D]. Consistent with these findings, heatmap analysis of the top 50 most regulated lipid species revealed overall constrained lipid profiles in chemoresistant cells [Supplementary Figure 6E and F].

### Therapeutic targeting and clinical validation identify context-dependent metabolic adaptations associated with chemoresistance in HR+/HER2- BC

**Figure 7:**
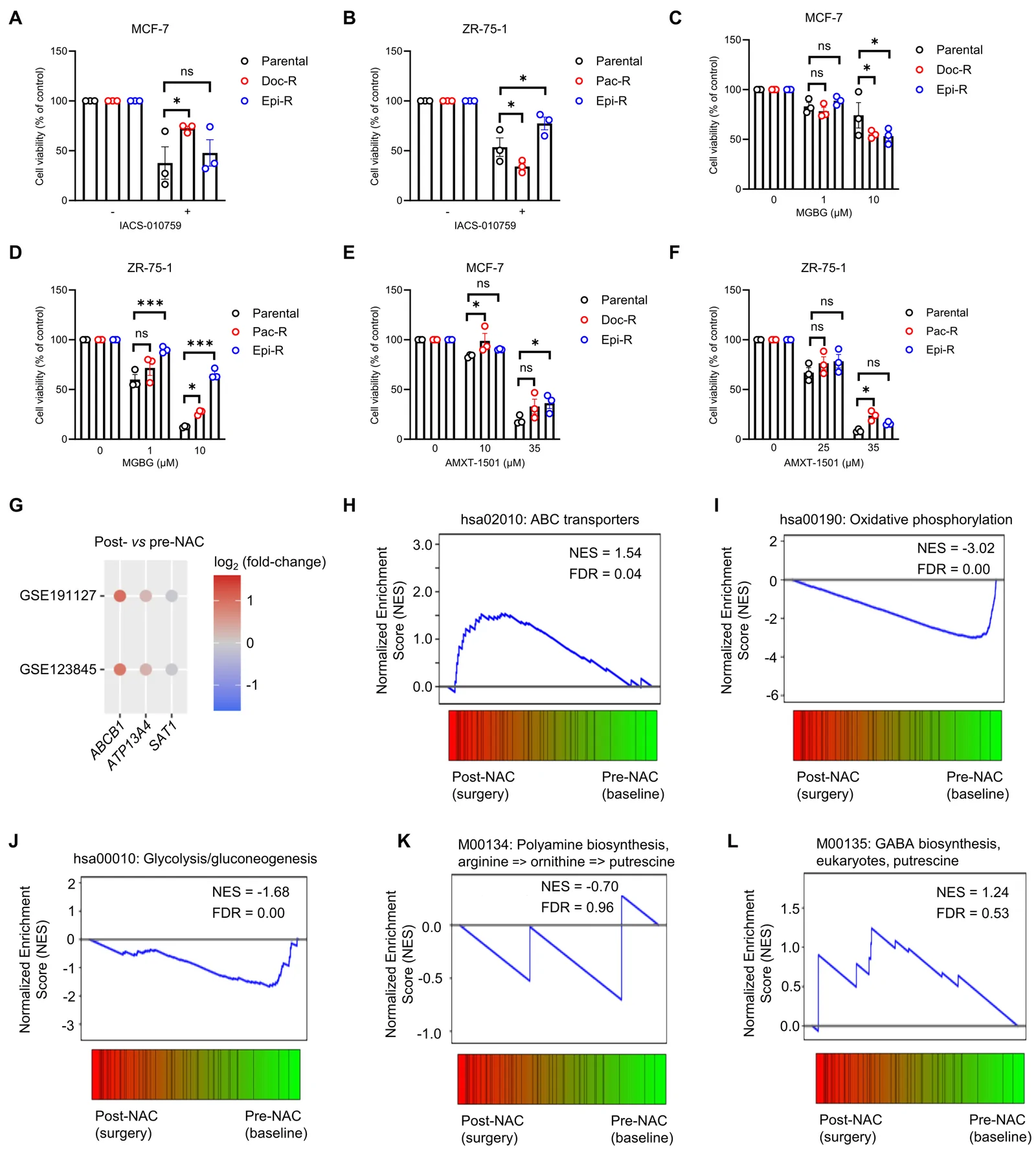
Therapeutic targeting and clinical validation identify context-dependent metabolic adaptations associated with chemoresistance in HR+/HER2- BC. (A and B) Viability of parental and chemoresistant MCF-7 (A) and ZR-75-1 (B) cells following 72 h of treatment with IACS-010759 (1 and 0.005 µM, respectively); (C and D) Viability of parental and chemoresistant MCF-7 (C) and ZR-75-1 (D) cells following 72 h of treatment with mitoguazone (MGBG); (E and F) Viability of parental and chemoresistant MCF-7 (E) and ZR-75-1 (F) cells following 72 h of treatment with AMXT-1501; (G) Bubble plot showing the log_2_ fold changes in *ABCB1*, *ATP13A4*, and *SAT1* gene expression in post- *vs.* pre-NAC samples from patients with HR+/HER2- BC in the GSE191127 and GSE123845 RNA-sequencing datasets. Color indicates the direction and magnitude of the log_2_ fold change; (H-L) Gene set enrichment analysis of post-NAC (surgery) *vs.* pre-NAC (baseline) samples from the GSE191127 dataset for ABC transporters (H), OXPHOS (I), glycolysis/gluconeogenesis (J), polyamine biosynthesis from arginine to putrescine (K), and GABA biosynthesis involving putrescine catabolism (L). Normalized enrichment scores (NES) and FDRs are indicated for each pathway. Data are presented as mean ± SEM from three independent biological experiments (*n* = 3), each performed with three technical replicate wells per condition (A-F). Individual data points represent the mean of the technical replicates from each independent biological experiment. Statistical significance was determined using two-way ANOVA with Sidák’s multiple comparison test (A-F). ^*^P < 0.05; ^***^P < 0.001; ns: not significant. HR+/HER2-: Hormone receptor-positive/human epidermal growth factor receptor 2-negative; BC: breast cancer; MGBG: methylglyoxal-bis(guanylhydrazone); NAC: neoadjuvant chemotherapy; ABC: ATP-binding cassette; OXPHOS: oxidative phosphorylation; GABA: γ-aminobutyric acid; NESs: normalized enrichment scores; FDRs: false discovery rates; SEM: standard error of the mean; ANOVA: analysis of variance; Doc-R: docetaxel-resistant; Epi-R: epirubicin-resistant; Pac-R: paclitaxel-resistant; *ABCB1*: ATP binding cassette subfamily B member 1; *SAT1*: spermidine/spermine N1-acetyltransferase 1.

To functionally assess metabolic dependencies, parental and chemoresistant MCF-7 and ZR-75-1 cells were treated with pharmacological inhibitors targeting mitochondrial respiration and polyamine metabolism. Inhibition of electron transport chain (ETC) complex I using IACS-010759 reduced cell viability across all models [Figure 7A and B]. However, contrary to our initial hypothesis, chemoresistant cells were overall less sensitive to treatment than their matching parental counterparts. This reduced sensitivity was particularly pronounced in MCF-7 Doc-R and ZR-75-1 Epi-R cells, in line with their decreased mitochondrial respiratory activity. Similarly, treatment with antimycin A, an ETC complex III inhibitor, decreased viability in both parental and resistant cells, with chemoresistant lines again exhibiting lower overall sensitivity [Supplementary Figure 7A and B]. These findings indicate that the diminished mitochondrial function observed in resistant cells translates into a reduced dependence on OXPHOS for survival.

We next evaluated polyamine metabolism using mitoguazone (MGBG), an inhibitor of S-adenosyl-methionine decarboxylase. In MCF-7 models, both Doc-R and Epi-R cells exhibited increased sensitivity to 10 µM MGBG compared with parental cells [Figure 7C]. In contrast, ZR-75-1 chemoresistant derivatives (Pac-R and Epi-R) were less affected by MGBG treatment than their parental counterparts [Figure 7D], indicating reduced sensitivity to polyamine biosynthesis inhibition. Similar results were obtained using DFMO, an ornithine decarboxylase inhibitor. In MCF-7 models, both Doc-R and Epi-R cells exhibited increased sensitivity compared with parental cells [Supplementary Figure 7C], whereas no significant differences were observed between parental and chemoresistant ZR-75-1 cells [Supplementary Figure 7D]. Finally, inhibition of polyamine transport using AMXT-1501 did not reveal consistent differential sensitivity between parental and chemoresistant cells in either MCF-7 or ZR-75-1 models [Figure 7E and F]. While MCF-7 Epi-R and ZR-75-1 Pac-R cells showed a modest trend toward reduced sensitivity at selected concentrations, these differences were minor and inconsistent across resistant sublines. Collectively, these findings reveal marked cell line-dependent divergence in responses to polyamine pathway inhibition. While chemoresistant MCF-7 cells displayed increased sensitivity to polyamine biosynthesis inhibitors (MGBG and DFMO), ZR-75-1 resistant cells did not exhibit enhanced sensitivity to either biosynthesis or transport inhibition (AMXT-1501).

To assess the translational relevance of these metabolic alterations, we analyzed pre- and post-NAC RNA-sequencing datasets from patients with HR+/HER2- BC (GSE191127 and GSE123845). *ABCB1* gene expression was increased following treatment in both clinical cohorts, whereas *ATP13A4* showed a more modest elevation and *SAT1* expression remained largely unchanged [Figure 7G]. Consistent with these transcriptomic shifts, gene set enrichment analysis revealed significant positive enrichment of the ABC transporter pathway in post-treatment residual tumors from GSE191127, with a similar positive trend observed in GSE123845 [Figure 7H and Supplementary Figure 7E]. In contrast, OXPHOS signature was significantly negatively enriched following chemotherapy in both clinical datasets [Figure 7I and Supplementary Figure 7F]. Glycolysis/gluconeogenesis pathway expression was likewise significantly negatively enriched in GSE191127, with GSE123845 showing a comparable but non-significant negative trend [Figure 7J and Supplementary Figure 7G]. Finally, neither the arginine-to-putrescine biosynthesis module nor the putrescine catabolism-related GABA biosynthesis module reached statistical significance in either patient cohort, although both exhibited a positive enrichment trend following treatment [Figure 7K and L, Supplementary Figure 7H and I].

Collectively, these findings demonstrate that chemoresistant HR+/HER2- BC cells adopt a metabolically constrained state characterized by reduced reliance on mitochondrial respiration and context-dependent vulnerabilities to polyamine pathway inhibition. Crucially, interrogation of clinical HR+/HER2- BC datasets corroborated the core metabolic features identified in our experimental models, including increased ABC transporter expression alongside the suppression of OXPHOS and glycolytic programs following NAC. In contrast, polyamine pathway alterations were less consistently detected in patient samples, supporting the concept that this metabolic adaptation is heterogeneous and defines context-specific rather than universal therapeutic vulnerabilities.

## DISCUSSION

NAC remains a cornerstone in the management of high-risk HR+/HER2- BC, yet many tumors exhibit incomplete pathological responses and eventually relapse. Defining the adaptive mechanisms that enable tumor cell survival under anthracycline and taxane exposure is therefore critical for improving therapeutic outcomes. A central finding of this study is the uncoupling between marked transcriptional plasticity and relative metabolic constraint. While RNA sequencing revealed extensive and largely non-overlapping gene expression changes, metabolomic and functional analyses converged toward relatively stable metabolic states. Chemoresistant cells exhibited reduced mitochondrial respiration without compensatory glycolytic activation, resulting in an overall low-bioenergetic phenotype. This contrasts with classical models of metabolic rewiring described in other BC subtypes, particularly triple negative disease, where resistance has been associated with increased glycolysis^[18,29,30]^ or enhanced OXPHOS^[16,31,32]^. Lipidomic analyses revealed predominantly reduced and heterogeneous lipid states, without strong convergence across models. While lipid remodeling has been implicated in chemoresistance^[33]^, our data suggest that, in HR+/HER2- BC, these alterations are more context-dependent and likely play a modulatory rather than central role.

Our findings instead support an alternative model in which resistance emerges through a low-flux, energy-conserving state, reminiscent of drug-tolerant persister cells^[34]^. In the absence of a classical compensatory shift toward glycolysis to offset impair OXPHOS, these cells must utilize alternative survival strategies to maintain viability under strict bioenergetic constraints. Rather than driving active proliferation, alternative metabolic networks, such as fatty acid oxidation or amino acid catabolism, may be strategically prioritized to meet minimal maintenance thresholds. Furthermore, such a low-flux state may be highly dependent on enhanced autophagy flux to supply critical metabolic intermediates and to sustain basal ATP levels and cellular homeostasis without necessitating high external nutrient uptake. Clinically, such a restrained metabolic state may contribute to the well-known propensity of HR+/HER2- BC to relapse many years after initial treatment^[35]^, consistent with the persistence of dormant, low-proliferative tumor cells. This contrasts with triple-negative BC, which more commonly displays early relapse kinetics^[36]^. Importantly, our interrogation of publicly available RNA-sequencing datasets from HR+/HER2- BC patients treated with NAC provided crucial clinical corroboration of this low-bioenergetic state. In residual post-treatment tumors, we observed significant negative enrichment of gene signatures associated with OXPHOS as well as glycolysis/gluconeogenesis. Simultaneously, classical drug resistance mechanisms were strongly maintained in patient samples, as demonstrated by the marked post-treatment upregulation of *ABCB1* expression and positive enrichment of ABC transporter signatures. These clinical observations demonstrate that the suppression of core bioenergetic pathways accompanied by drug efflux engagement is not merely an *in vitro* artifact, but a cardinal feature of residual, chemoresistant HR+/HER2- tumors in patients.

Despite this global bioenergetic suppression, selective metabolic adaptations were identified. Polyamine metabolism emerged as a recurrent but heterogeneous feature across resistant models, with increased spermidine and spermine levels and upregulation of ATP13A4 and SAT1, particularly in anthracycline-resistant models. Crucially, polyamines are well-established upstream regulators of stress adaptation and are intimately linked to the activation of cytoprotective autophagy^[37,38]^. In our models, altered polyamine homeostasis may directly interface with this autophagic machinery, reinforcing macromolecular recycling to preserve cell survival beneath standard bioenergetic thresholds. Polyamines have also been shown to promote mitophagy, improving mitochondrial quality control while limiting mitochondrial ROS production^[39]^. In parallel, spermidine-dependent hypusination of eIF5A supports the translation of key mitochondrial proteins involved in β-oxidation and oxidative metabolism, thereby preserving a functional mitochondrial network and the minimal metabolic activity required for cell survival^[40]^. Their involvement in therapy resistance has been reported in several tumoral contexts, including BC and melanoma, where elevated polyamine levels promote survival under genotoxic and oxidative stress^[41,42]^. In the context of reduced bioenergetic activity, altered polyamine homeostasis may contribute to the maintenance of cell survival under cytotoxic stress.

Functionally, our data indicate a cell line-dependent divergence in metabolic dependencies. In MCF-7 models, chemoresistant cells (Doc-R and Epi-R) exhibited increased sensitivity to polyamine biosynthesis inhibitors, whereas no differential sensitivity was observed following inhibition of polyamine transport, despite elevated ATP13A4 expression in anthracycline-resistant cells. In contrast, chemoresistant ZR-75-1 cells (Pac-R and Epi-R) showed no increased sensitivity to either polyamine biosynthesis or transport inhibition compared with their parental counterparts. These findings indicate that alterations in polyamine metabolism do not translate into a uniform therapeutic vulnerability across resistant HR+/HER2- BC cells. Rather, dependencies on polyamine homeostasis appear to be context-dependent and influenced by both cellular background and the specific metabolic node targeted. The differential responses observed between biosynthesis and transport inhibition further suggest that resistant cells may engage distinct compensatory mechanisms to maintain polyamine balance. Similar adaptive responses have been described in other cancer contexts and have motivated the development of combination strategies targeting both polyamine synthesis and transport^[43-45]^. Polyamine homeostasis is all the more critical because it must be maintained within a tight physiological range; a decrease in polyamines disrupts pathways essential for growth and survival, while excessive accumulation can trigger compensatory catabolic pathways [via spermine oxidase (SMOX)/peroxisomal N1-acetylpolyamine oxidase (PAOX)], generating hydrogen peroxide (H_2_O_2_) and oxidative stress that can have deleterious effects^[46]^, paving the way for several anticancer strategies targeting polyamines. The heterogeneity of polyamine alterations in our experimental models was mirrored in the clinical datasets. While *ATP13A4* expression and polyamine biosynthesis signatures showed positive enrichment trends in post-treatment patient samples, these changes were less uniform and did not reach statistical significance across all cohorts. This clinical variability underscores that, unlike the consistent suppression of central bioenergetics and induction of drug transporters, polyamine pathway remodeling represents a patient- or context-specific adaptation rather than a universal clinical hallmark.

More broadly, our results support the growing interest in metabolic profiling for precision oncology. Distinct metabolic subtypes have been identified in BC and are associated with differential therapeutic responses and clinical outcomes^[47,48]^. In this context, integrating metabolic features with transcriptomic and functional data may help refine patient stratification and identify clinically relevant vulnerabilities, particularly in residual disease after chemotherapy.

This study has limitations. It relies on *in vitro* models and analyses of bulk transcriptomic data from clinical tumor cohorts, which do not fully capture the complexity of the tumor microenvironment or systemic metabolic interactions *in vivo*. In addition, the multi-omics approach is primarily descriptive and does not establish causal relationships between specific metabolic alterations and chemoresistance. However, this integrative strategy represents a key strength, as it allows discrimination between highly heterogeneous, context-specific changes and recurrent adaptations, thereby highlighting pathways of potential functional relevance.

In conclusion, acquired resistance to anthracyclines and taxanes in HR+/HER2- BC is characterized by transcriptional divergence but metabolic constraint, with selective and context-dependent alterations in mitochondrial and polyamine metabolism. Rather than supporting a universal metabolic phenotype, our findings indicate that resistant cells engage distinct adaptive strategies depending on cellular background and therapeutic pressure, offering a clear framework for the development of tailored, metabolism-based strategies to overcome therapeutic resistance in HR+/HER2- BC^[49]^.

## DECLARATIONS

### Availability of data and materials

Datasets from RNA-sequencing analyses, generated during this study, are available at Gene Expression Omnibus: GSE327278 (parental and Doc-R MCF-7 cells), GSE328653 (parental, Pac-R ZR-75-1, Epi-R ZR-75-1, and Epi-R MCF-7 cells). All unique reagents generated in this study will be made available on request to Prof. Cyril Corbet (cyril.corbet@uclouvain.be), with a completed material transfer agreement.

### AI and AI-assisted tools statement

During the preparation of this manuscript, the AI tool Gemini (Advanced version, released 2026-02-15) was used solely for language editing. The tool did not influence the study design, data collection, analysis, interpretation, or the scientific content of the work. All authors take full responsibility for the accuracy, integrity, and final content of the manuscript.

### Copyright

© The Author(s) 2026.
