# Supplementary material for: Divergent transcriptional programs and constrained metabolic states reveal selective metabolic dependencies in chemoresistant luminal breast cancer
Source: Cancer Drug Resist. 2026 Aug 28;9:30. doi: 10.20517/cdr.2026.57 (PMC13563457; doi:10.20517/cdr.2026.57)
Supplement: Supplementary file 1 [file cdr-9-30-SupplementaryMaterials.pdf]

Supplementary Materials

Divergent transcriptional programs and constrained metabolic states reveal selective metabolic dependencies in chemoresistant luminal breast cancer

Melis Poyraz[1], Buse Sari[1], Jérôme Ambroise[2], Melanie Spears[3,4], Martin Michaelis[5,6], Jindrich Cinatl Jr.[6], Romain Boidot[7], Kristian Serafimov[8], Olivier Feron[1,9], François P. Duhoux[10,11], Manon Desgres[1,#], Cyril Corbet[1,9,#]

[1]Pole of Pharmacology and Therapeutics (FATH), Institut de Recherche Expérimentale et Clinique (IREC), UCLouvain, Brussels 1200, Belgium.
[2]Centre des Technologies Moléculaires Appliquées (CTMA), Institut de Recherche Expérimentale et Clinique (IREC), UCLouvain, Brussels 1200, Belgium.
[3]Diagnostic Development, Ontario Institute for Cancer Research, Toronto M5G 1M1, Canada.
[4]Department of Laboratory Medicine and Pathobiology, University of Toronto, Toronto M5S 3K9, Canada.
[5]School of Natural Sciences, Stacey Building, University of Kent, Canterbury CT2 7NJ, UK.
[6]Dr Petra Joh-Research Institute, Frankfurt am Main 60528, Germany.
[7]Université Bourgogne Europe, Centre Georges-François Leclerc, Unicancer, Unit of Molecular Biology, Department of Biology and Pathology of Tumors, ICMUB UMR CNRS 6302, Dijon 21000, France.
[8]IREC Metabolomics Core, UCLouvain, Brussels 1200, Belgium.
[9]WEL Research Institute, Wavre 1300, Belgium.
[10]Pole of Molecular Imaging, Radiotherapy and Oncology (MIRO), Institut de Recherche Expérimentale et Clinique (IREC), UCLouvain, Brussels 1200, Belgium.
[11]Institut Roi Albert II, Department of Medical Oncology, Cliniques universitaires Saint-Luc, Brussels 1200, Belgium.
[#]These authors equally supervised the work.

Correspondence to: Prof. Cyril Corbet, Pole of Pharmacology and Therapeutics (FATH), Institut de Recherche Expérimentale et Clinique (IREC), UCLouvain, Brussels 1200, Belgium. E-mail: cyril.corbet@uclouvain.be

Inventory of Supplementary Materials:

Supplementary Figure 1: Extended characterization of chemoresistant phenotypes in HR+/HER2- BC models.
Supplementary Figure 2: Transcriptomic analyses highlight limited overlap between drug- and cell line-specific gene expression changes in chemoresistant HR+/HER2- BC cells.
Supplementary Figure 3: Validation of drug-specific gene expression changes in anthracycline- and taxane-resistant HR+/HER2- BC cells.
Supplementary Figure 4: Glucose consumption and lactate secretion are not increased in chemoresistant HR+/HER2- BC cells.
Supplementary Figure 5: Metabolomic profiling reveals limited global separation between parental and chemoresistant HR+/HER2- BC cells.
Supplementary Figure 6: Lipidomic profiling reveals greater global divergence between parental and chemoresistant HR+/HER2- BC cells.
Supplementary Figure 7: Therapeutic targeting and clinical validation identify context-dependent metabolic adaptations associated with chemoresistance in HR+/HER2- BC.
Supplementary Table 1. Sequences of human gene-specific primers for qRT-PCR used in this study.
Supplementary Methods

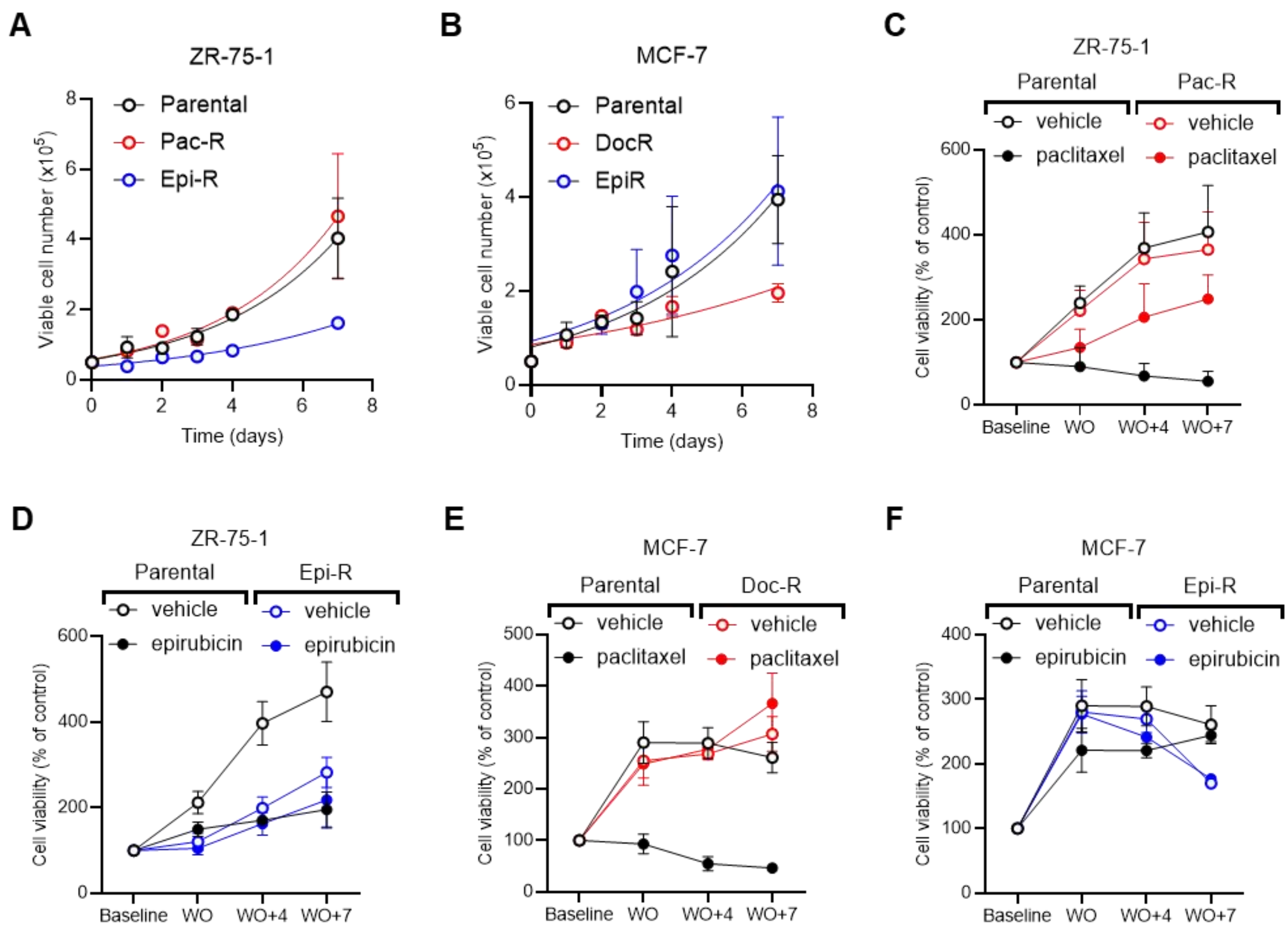

Supplementary Figure 1. Extended characterization of chemoresistant phenotypes in HR+/HER2- BC models. (A-B) Growth kinetics of parental and chemoresistant ZR-75-1 (A) and MCF-7 (B) cells monitored over 7 days under standard culture conditions. (C-D) Viability of parental and chemoresistant ZR-75-1 cells measured at baseline, after 72 h of treatment with 25 nM paclitaxel (C) or 10 nM epirubicin (D) (WO), and during the recovery phase at 4 and 7 days post-washout (WO+4 and WO+7). (E-F) Viability of parental and chemoresistant MCF-7 cells measured at baseline, after 72 h of treatment with 25 nM paclitaxel (E) or 30 nM epirubicin (F) (WO), and during the recovery phase at 4 and 7 days post-washout (WO+4 and WO+7). Data are presented as mean ± SEM from three independent biological experiments (n=3), each performed with three technical replicate wells per condition (A-F). Individual data points represent the mean of the technical replicates from each independent biological experiment.

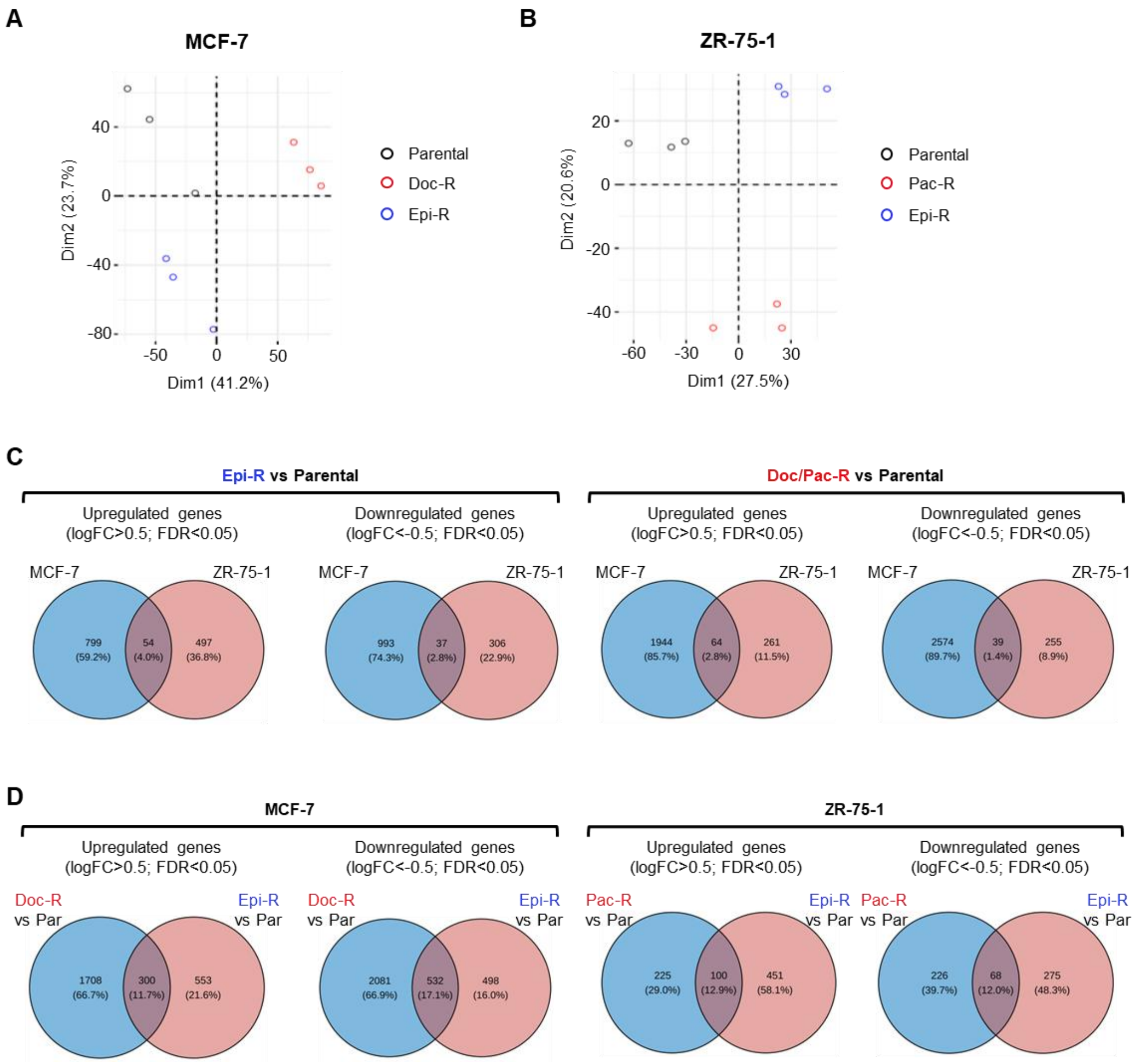

Supplementary Figure 2. Transcriptomic analyses highlight limited overlap between drug- and cell line-specific gene expression changes in chemoresistant HR+/HER2- BC cells. (A-B) Principal component analysis plots of parental and chemoresistant MCF-7 (A) and ZR-75-1 (B) cells based on RNA sequencing data. (C) Venn diagrams illustrating the overlap of drug-specific differentially expressed genes (logFC<-0.5 or >0.5; FDR<0.05) in taxane- and anthracycline-resistant HR+/HER2- BC cell models. (D) Venn diagrams illustrating the overlap of cell line-specific differentially expressed genes (logFC<-0.5 or >0.5; FDR<0.05) in taxane- and anthracycline-resistant HR+/HER2- BC cell models.

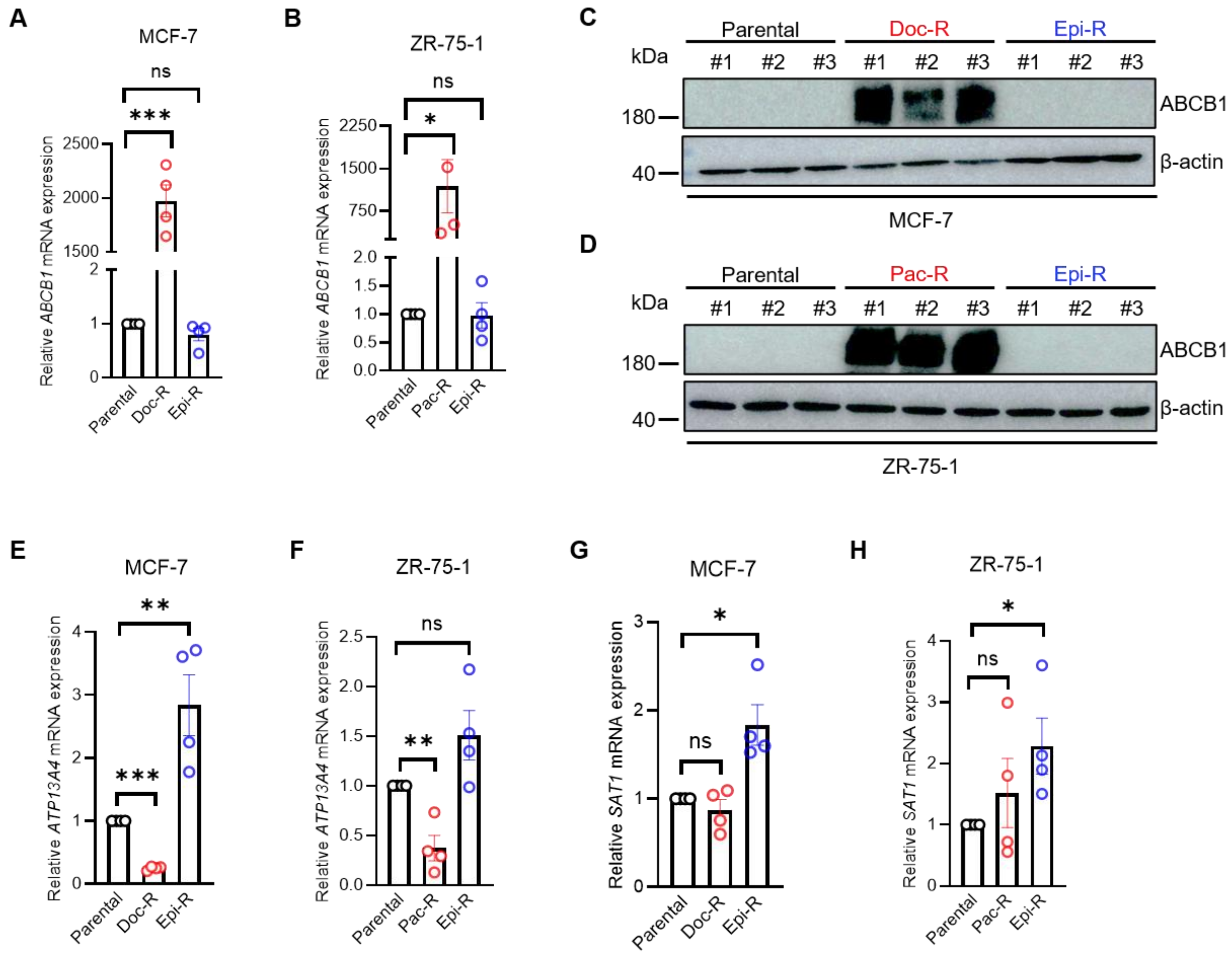

Supplementary Figure 3. Validation of drug-specific gene expression changes in anthracycline- and taxane-resistant HR+/HER2- BC cells. (A–B) Relative *ABCB1* mRNA expression in parental and chemoresistant MCF-7 (A) and ZR-75-1 (B) cells. (C-D) Representative immunoblots showing ABCB1/MDR1 protein expression in parental and chemoresistant MCF-7 (C) and ZR-75-1 (D) cells. (E–H) Relative mRNA expression of *ATP13A4* (E-F) and *SAT1* (G-H) in parental and chemoresistant MCF-7 and ZR-75-1 cells. For Western blot analyses, samples labeled #1, #2, and #3 represent three independent biological replicates (n=3) prepared from independently cultured and harvested cells (C-D). For qRT-PCR analyses, data are presented as mean ± SEM from four independent biological experiments (n=4), with individual data points representing distinct biological replicates (A-B and E-H). Statistical significance was determined using an unpaired Student's *t*-test (A-B and E-H). **P*<0.05; ***P*<0.01; ****P*<0.001; ns, not significant.

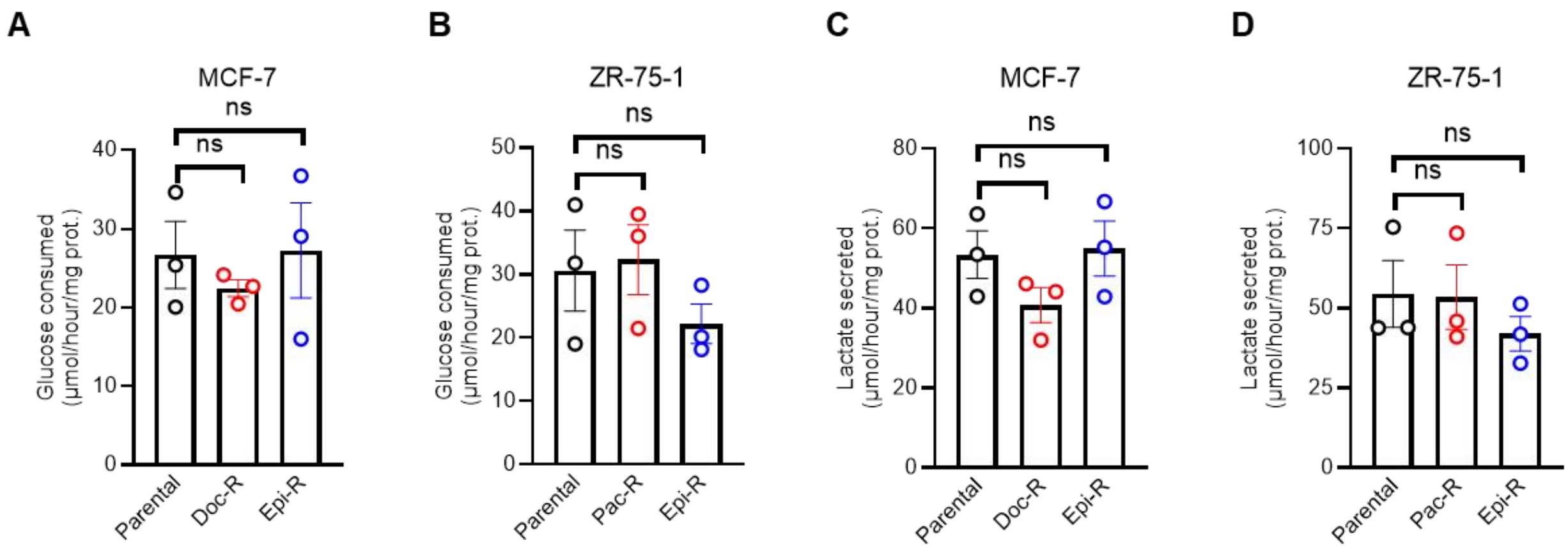

Supplementary Figure 4. Glucose consumption and lactate secretion are not increased in chemoresistant HR+/HER2- BC cells. (A-B) Glucose consumption in parental and chemoresistant MCF-7 (A) and ZR-75-1 (B) cells. (C-D) Lactate secretion in parental and chemoresistant MCF-7 (C) and ZR-75-1 (D) cells. Data are presented as mean ± SEM from three independent biological experiments (n=3), each performed with three technical replicate wells per condition (A-D). Individual data points represent the mean of the technical replicates from each independent biological experiment. Statistical significance was determined using one-way ANOVA with Dunnett's multiple comparison test (A-D). ns, not significant.

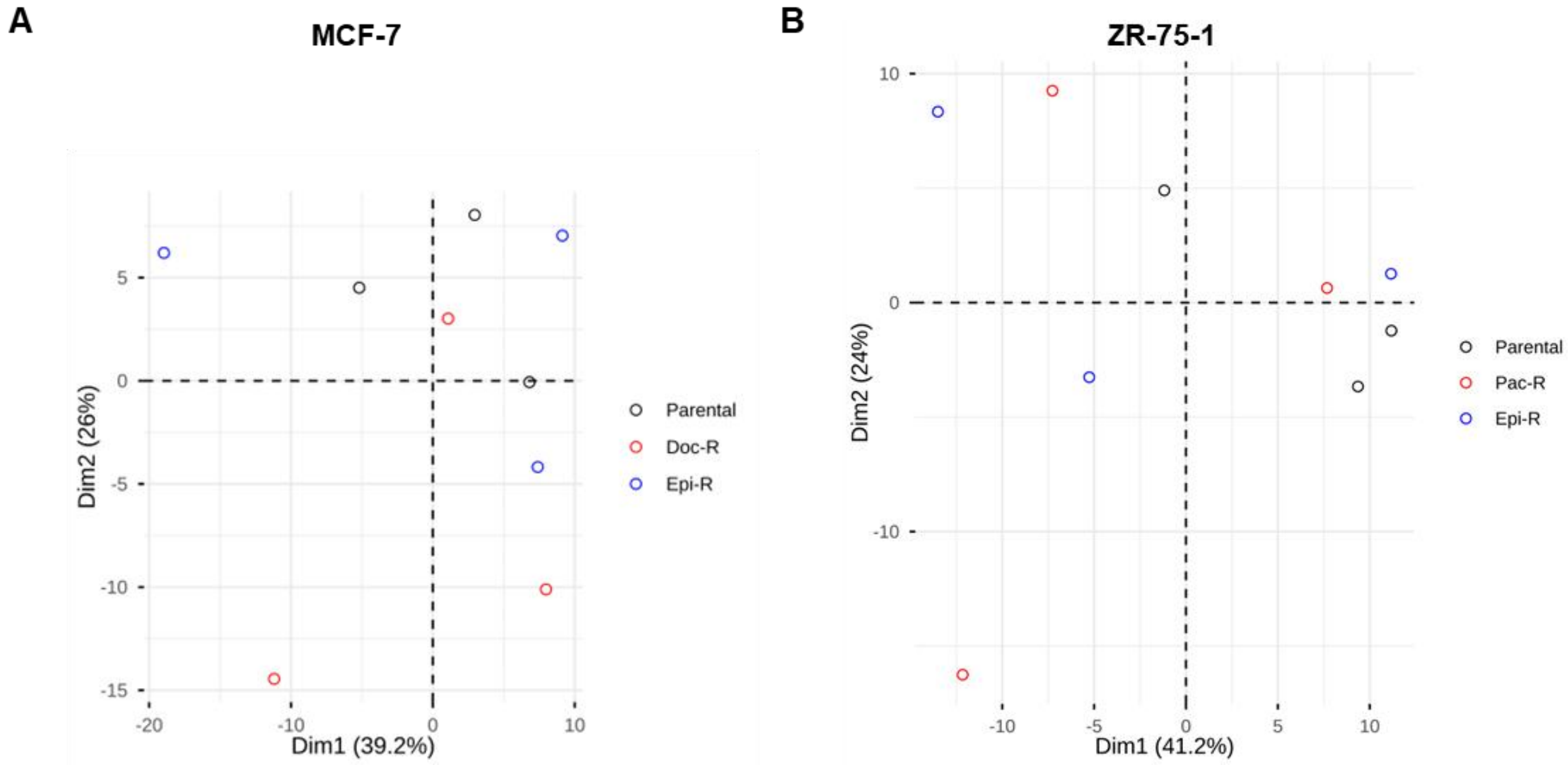

Supplementary Figure 5. Metabolomic profiling reveals limited global separation between parental and chemoresistant HR+/HER2- BC cells. (A-B) Principal component analysis plots of parental and chemoresistant MCF-7 (A) and ZR-75-1 (B) cells based on metabolomics data.

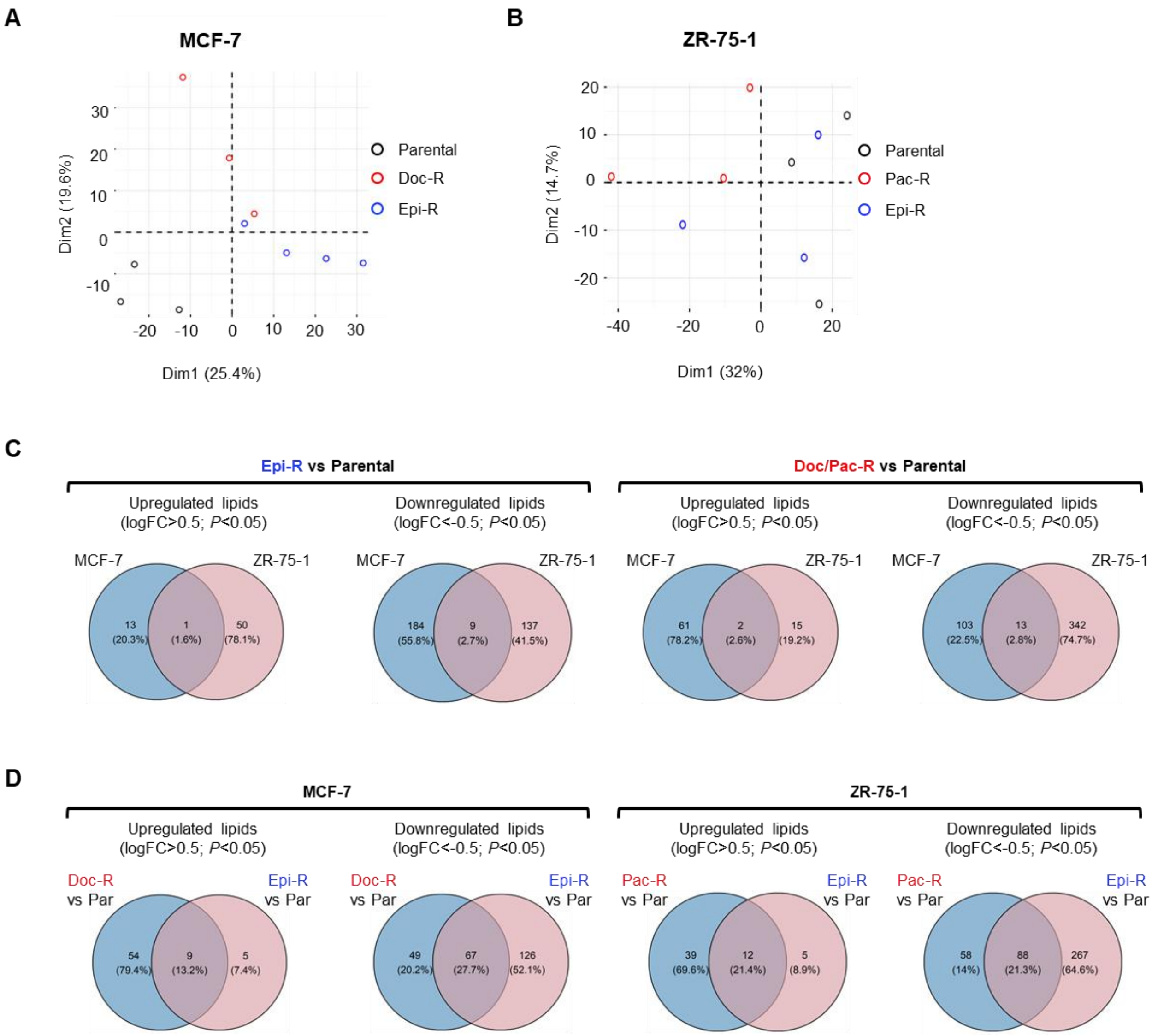
A
MCF-7
Dim2 (19.6%)
Dim1 (25.4%)
Parental
Doc-R
Epi-R
B
ZR-75-1
Dim2 (14.7%)
Dim1 (32%)
Parental
Pac-R
Epi-R
C
Epi-R vs Parental
Doc/Pac-R vs Parental
Upregulated lipids (logFC>0.5; P<0.05)
Downregulated lipids (logFC<-0.5; P<0.05)
MCF-7
ZR-75-1
13 (20.3%)
1 (1.6%)
50 (78.1%)
184 (55.8%)
9 (2.7%)
137 (41.5%)
61 (78.2%)
2 (2.6%)
15 (19.2%)
103 (22.5%)
13 (2.8%)
342 (74.7%)
D
MCF-7
ZR-75-1
Doc-R vs Par
Epi-R vs Par
Pac-R vs Par
54 (79.4%)
9 (13.2%)
5 (7.4%)
49 (20.2%)
67 (27.7%)
126 (52.1%)
39 (69.6%)
12 (21.4%)
5 (8.9%)
58 (14%)
88 (21.3%)
267 (64.6%)

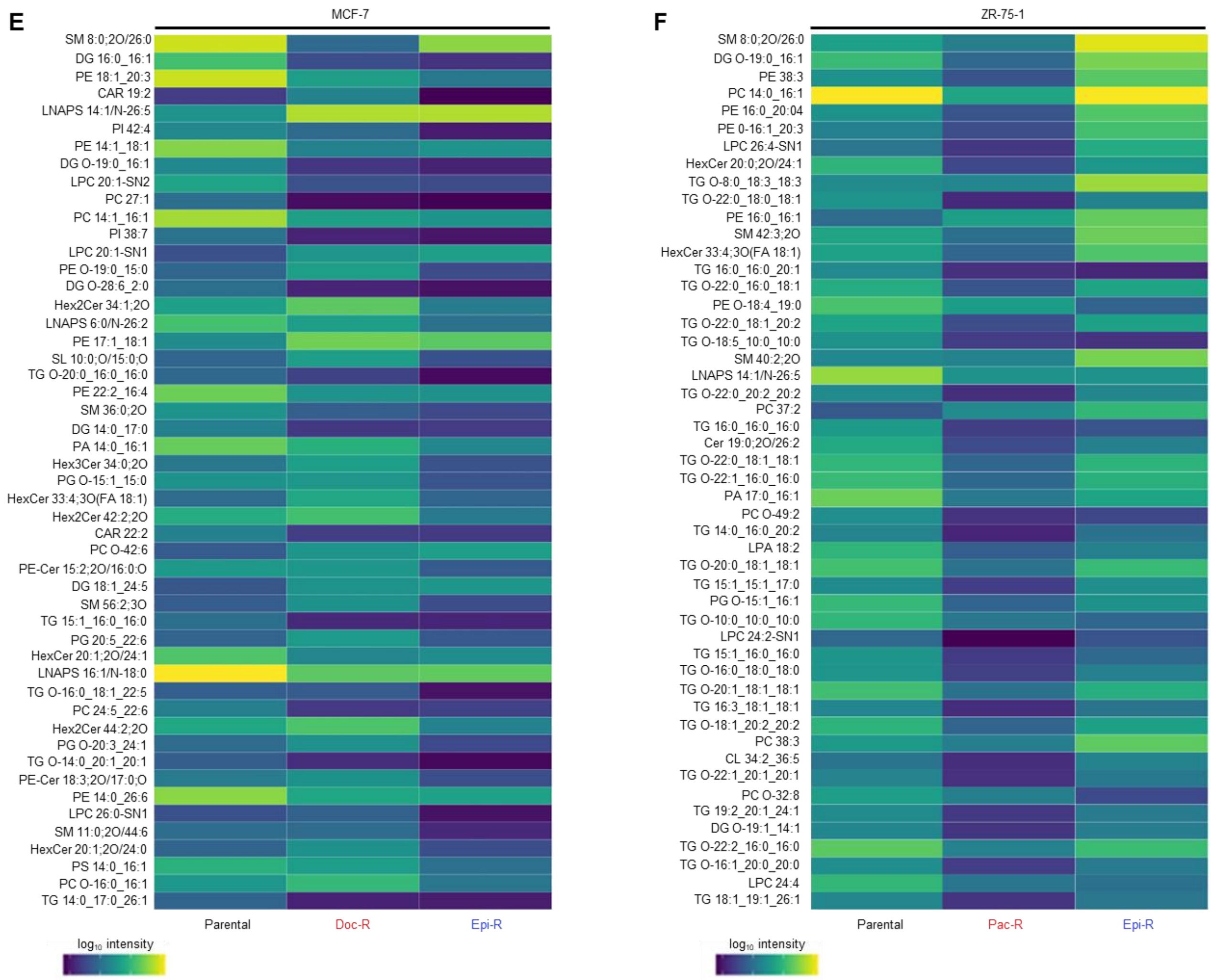

Supplementary Figure 6. Lipidomic profiling reveals increased divergence between parental and chemoresistant HR+/HER2- BC cells. (A-B) Principal component analysis of lipidomic profiles in parental and chemoresistant MCF-7 (A) and ZR-75-1 (B) cells. (C) Venn diagrams showing the overlap of drug-specific differentially abundant lipid species (logFC<-0.5 or >0.5; *P*<0.05) between taxane- and anthracycline-resistant HR+/HER2- BC cell models. (D) Venn diagrams showing the overlap of cell line-specific differentially abundant lipids species (logFC<-0.5 or >0.5; *P*<0.05) between taxane- and anthracycline-resistant HR+/HER2- BC cell models. (E-F) Heatmaps of the top 50 most regulated lipid species in chemoresistant MCF-7 (E) and ZR-75-1 (F) models relative to their parental counterparts. Color scale represents log10-transformed intensities.

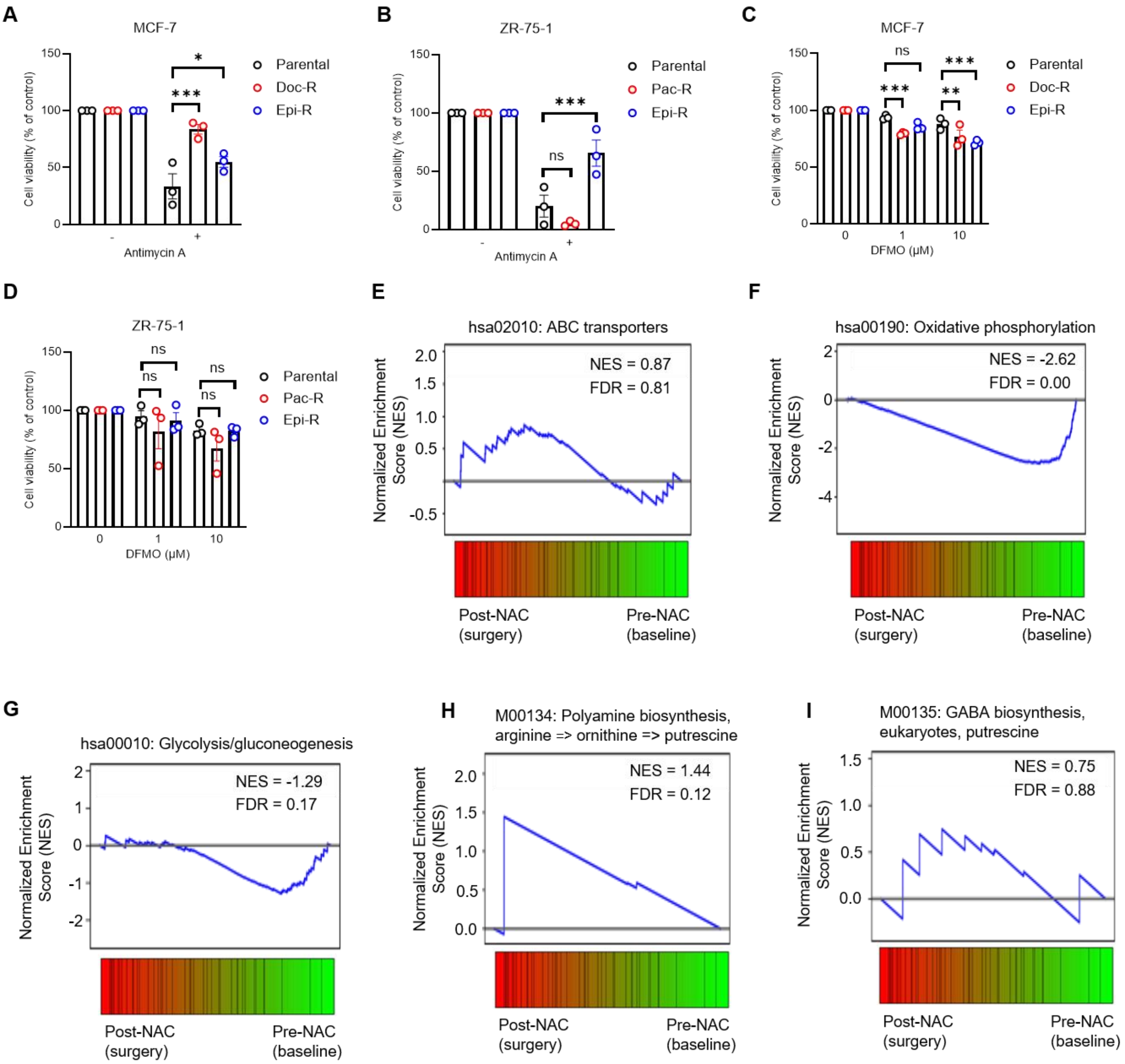

Supplementary Figure 7. Therapeutic targeting and clinical validation identify context-dependent metabolic adaptations associated with chemoresistance in HR+/HER2- BC. (A-B) Viability of parental and chemoresistant MCF-7 (A) and ZR-75-1 (B) cells following 72 h of treatment with 0.6 µM antimycin A. (C-D) Viability of parental and chemoresistant MCF-7 (C) and ZR-75-1 (D) cells following 72 h of treatment with DFMO. (E-I) Gene set enrichment analysis of post-NAC (surgery) versus pre-NAC (baseline) samples from the GSE123845 dataset for ABC transporters (E), OXPHOS (F), glycolysis/gluconeogenesis (G), polyamine biosynthesis from arginine to putrescine (H), and GABA biosynthesis involving putrescine catabolism (I). Normalized enrichment scores (NES) and false discovery rates (FDR) are indicated for each pathway. Data are presented as mean ± SEM from three independent biological experiments (n=3), each performed with three technical replicate wells per condition (A-D). Individual data points represent the mean of the technical replicates from each independent biological experiment. Statistical significance was determined using two-way ANOVA with Sidák's multiple comparison test (A-D). **P*<0.05; ***P*<0.01; ****P*<0.001; ns, not significant.

Supplementary Table 1. Sequences of human gene-specific primers for qRT-PCR used in this study

Gene name	Forward primer (5' - 3')	Reverse primer (5' - 3')	Amplicon length (bp)
ABCB1	TGACAGCTACAGCACGGAAG	TCTTCACCTCCAGGCTCAGT	131
ATP13A4	CCAGCACGCTCTGCTCAATG	GAAGATGGATCCGGCAAGGC	103
GTF2B	ACTACAGAGCCGGTGATATGAT	GTTGCTTTGTCATTGCTGAAAGT	106
SAT1	CCGTGGATTGGCAAGTTATT	TCCAACCCTCTTCACTGGAC	217

Supplementary Methods

Metabolomics analysis

Chemicals and reagents

Formic acid, acetic acid, and acetonitrile (Ultra LC-MS grade) were obtained from Fisher Scientific (Brussels, Belgium). Ammonium hydroxide solution (Suprapur®, 25% NH_3 basis) and N-ethylmaleimide were purchased from Sigma-Aldrich (Merck, Hoeilaart, Netherlands). Stable isotope-labeled metabolite internal standards were obtained from Buchem BV (Apeldoorn, Netherlands) and Sanbio (Uden, Netherlands), including labeled amino acids, organic acids, and nucleotide standards. PFA bottles for mobile phases were provided by AHF Analysentechnik (Tübingen, Germany).

Metabolite extraction

Cell samples were transferred to homogenization tubes. One milliliter of extraction solvent (50:50 MeOH/H_2O v/v; methanol containing 20 mM N-ethylmaleimide) was added together with 50 µL of internal standard mixture and 0.15 g zirconia/glass beads. Samples were homogenized at 4°C for 10 cycles using a Precellys Evolution homogeneizer (10 cycles, 10 s each, 5,000 rpm). Lysates were centrifuged (20,000 x g, 5 min, 4°C), and the supernatant was collected. Derivatization was stopped by adding 10 µL of 1 M ammonium formate buffer (pH 6.0). Extracts were evaporated to dryness and reconstituted in acetonitrile/H_2O (50:50; v/v).

LC-MS analysis

Metabolomic analyses were performed using a ThermoFisher Vanquish Horizon UHPLC system coupled to a high-resolution Orbitrap Exploris 240 mass spectrometer. Chromatographic separation was achieved using a BEH amide HILIC column (100 x 2.1 mm; 1.7 µm) maintained at 60°C. Data were acquired in both positive and negative electrospray ionization modes with full-scan acquisition and data-dependent MS/MS fragmentation across a mass range of 70-900 m/z.

Data processing

Data processing included peak detection, alignment, blank subtraction, and metabolite identification using MS-DIAL software (version 4.9.221218). Metabolite annotations were supported by retention time matching, accurate mass, isotopic pattern, and MS/MS spectral similarity against a curated in-house spectral library. Abundances were normalized using both cell counts and isotopically labeled internal standards.

Lipidomics analysis

Chemicals and reagents

LC-MS grade solvents including acetonitrile, water, isopropanol, and ammonium formate were obtained from Fisher Scientific (Brussels, Belgium). Lipid internal standards were purchased from Avanti Polar Lipids (Alabaster, AL, USA).

Lipid extraction

Lipids were extracted using a monophasic MeOH/IPA/MTBE (1:1:1.33 v/v/v) protocol in the presence of internal standards and butylated hydroxytoluene (BHT). Samples were ultrasonicated for 10 min and centrifuged (20,000 x g, 5 min, 4°C). Supernatants were dried under nitrogen and reconstituted in methanol prior to LC-MS analysis.

LC-MS analysis

Lipidomics analyses were performed using a ThermoFisher Vanquish Horizon UHPLC system coupled to an Orbitrap Exploris 240 mass spectrometer. Lipid species were separated on a Waters Acquity CSH C18 column (100 x 1.0 mm; 1.7 µm) using a binary gradient of aqueous and organic mobile phases containing formic acid and ammonium formate. Mass spectra were acquired in positive and negative ionization modes across a mass range of 100-1500 m/z with data-dependent MS/MS fragmentation.

Data processing

Peak integration and feature alignment were performed using MS-DIAL (version 4.9.221218) with additional manual inspection using Thermo FreeStyle software. Lipid identification was performed using an in-house spectral library, based on retention time, precursor mass, isotopic pattern, and MS/MS spectral similarity. Lipid abundances were normalized using cell number and internal standards before downstream statistical analysis in R and Python.
